# The Transfer Model and Guidance Strategy of Netizens' Emotions

**DOI:** 10.3389/fpsyg.2022.880322

**Published:** 2022-05-19

**Authors:** Zhitao Wen, Yixue Xia, Mo Liu, Yuexin Lan

**Affiliations:** Research Center for Network Public Opinion Governance, China People's Police University (CPPU), Langfang, China

**Keywords:** netizens' emotion, transfer model, guidance strategy, optimization, post-pandemic

## Abstract

In the context of the COVID-19 pandemic, a large amount of information is gathered on Internet platforms, through which people express their opinions and vent their emotions. Emotional guidance of netizens has become an important part of social governance during turbulences caused by a so-called “infodemic”. This study focuses on the evolution and interaction of netizens' emotions after the occurrence of network public opinion events. First, the transfer model of netizens' emotions is constructed, and the significance of each parameter in the model is studied through simulation. Then, based on the model, we put forward the optimization method and quantitative method of guidance strategy of netizens' emotions. Finally, the empirical study proves the effectiveness of the model, which can provide a theoretical basis for the emotional guidance strategies after the outbreak of network public opinion events.

## Introduction

Emotional contagion is common in the crowd. Each individual acts as an emotional sensor to affect others' emotions, and then affect others' judgment and behavior (Barsade, 2002). Under the COVID-19 pandemic, the characteristics of emotional contagion have been different from those in the normal environment. The global pandemic of COVID-19 has caused a lot of anxiety. In the face of the epidemic, some people were more vulnerable to experiencing anxiety and other mental health symptoms (Wheaton et al., 2021). Due to the segregated environments, people's cognition of happiness was significantly reduced, while their cognition of negative emotions was significantly improved (Meléndez et al., 2020), and the expectation of “return to normal” was a strong emotion under the pandemic (Slaughter et al., 2021). In short, compared with pre-pandemic, people's physical and psychological conditions have decreased significantly, and the important factor causing this decline was that it was difficult for people to describe and regulate emotions (Panayiotou et al., 2021). COVID-19 not only affected emotional infection but also changed people's social communication mode. In the context of the popularity of COVID-19, the control of governments restricts people's offline communication to a certain extent, and more social activities have moved online. Research showed that posting through social networks could also affect people's emotions and achieve large-scale transmission (Kramer et al., 2014), which provided an efficient way for emotional contagion. In addition, even without deliberately manipulating the posting of content in social networks, due to individual differences, netizens would show positive or negative emotions after receiving information (Ferrara and Yang, 2015), which showed that the dissemination of emotions on the Internet does not need deliberate behavior, but a spontaneous process. At the same time, because the Internet could efficiently spread information, emotional contagion was also accelerated on social networks, and the emotional synchronization of netizens may be strengthened (Coviello et al., 2014), which objectively strengthened the emotional contagion among netizens. It can be seen that emotional contagion among netizens is widespread, with the characteristics of large-scale and high-speed transmission, and this process is not limited by region.

It can be seen from the above analysis that under the COVID-19 pandemic and the prevalence of social networks when a focus event occurs in society, a large number of netizens' comments about the event will be posted on the social platform, which implies the emotional tendency of netizens—favorable comments often imply positive emotions and negative ones imply negative emotions. This process is also accompanied by emotional contagion among netizens. Therefore, the analysis of netizens' comment information essentially involves the analysis of netizens' emotions. Identifying and classifying the implicit emotion in the comment text is the basis of analyzing the emotion of netizens, and a large number of studies focus on it. Traditional machine learning methods such as SVM and Naive Bayes have been applied to multi-classification and prediction of text emotion (Kirange and Deshmukh, 2012; Krishnan et al., 2017). The development of artificial neural networks also promotes the research in this field and the language processing models such as RNN, LSTM, transformer, and Bert have been widely used (Zhang Q. et al., 2020; Banothu et al., 2021; Nemes and Kiss, 2021; Yang et al., 2021). On the basis of text emotion recognition and classification, some studies have begun to pay attention to the interaction between individual emotion and group emotion (Zeng and Zhu, 2019), the promotion of different emotions in information dissemination (Zhang L. et al., 2020), and the evolution and prediction of netizens' emotions (Jin and Yang, 2018; Ren et al., 2019). LDA model has been applied to study the relationship between topic words evolution (Tan et al., 2021), event topic (Zhou and Zhang, 2017), and netizen emotion. In addition, the mathematical models represented by SIR, SIS, SEIR model, and their improved model were also used to study the emotional contagion among netizens. Fang et al. constructed the S-SEIR Epidemic model based on netizens' emotions. This model considered the text's emotion and the transmission law of the text. The simulation experiment showed that text emotion could affect the transmission behavior of information (Fang et al., 2019). Considering the popularity of social media, Xu et al. constructed the time-varying SIR model to study the impact of social media on COVID-19 dissemination. The study found that the negative emotions on Twitter are related to a lower transmission rate of the corresponding susceptible compartment (Xu et al., 2021). Hill et al. (2010) assessed the spread of emotions in social networks through the SISa model and proved that both positive and negative emotions can exist in social networks for a long time like infectious diseases. The research on the spread of emotions in the crowd is also worthy of reference. Xiang et al. paid attention to the emotional contagion in dynamic people and simulated the emotional interaction in the process of crowd aggregation by the SIR model (Xiang et al., 2017a,b). Liu et al. focused on the factors that affect the values of emotions in the process of emotional contagion and constructed the SOSa–SPSa model. The simulation results showed that the values of positive emotion and negative emotion are positively correlated with spontaneity and contagion, and negatively correlated with recovery (Liu et al., 2014). Yi et al. studied the influence of emotion on information diffusion in online social networks (Yi et al., 2019).

The above studies generally focus on the following aspects: (1) identifying and classifying netizens' emotions through natural language processing technology; (2) research on the relationship between netizens' emotions and other elements such as public topics and Internet rumors; and (3) through mathematical modeling (most of them are based on SIR model and its improved model), studying the communication states of netizens' emotions, the factors affecting emotional contagion, and the impact of emotions on the dissemination of other information. However, few studies pay attention to the changes in netizens' emotions and the interaction between different emotions from the perspective of event evolution. By improving the logistic model, we studied the continuous evolution and interaction mechanism of netizens' emotions under a network public opinion event. The contributions of the model are as follows: (1) describing the evolution law of a single emotion over time when there is an interaction between multiple emotions; (2) different emotional interaction modes are simulated by changing the table of parameters; and (3) providing a quantitative way of netizens' emotional guidance and guidance effect, and carry out empirical research on the basis of the theoretical model, which proves the rationality of the theory proposed in this article.

## Model Establishment and Parameter Calculation

In this section, first, we explain the modeling method of the emotional information based on the logistic model. Then, we construct a logistic model considering the transfer of emotion and giving the meaning of each parameter and the solution of the equilibrium point. Finally, we explain the method of estimating model parameters by constructing difference equations and multiple linear regression analysis.

### The Original Model of Netizens' Emotions

**Figure 1:**
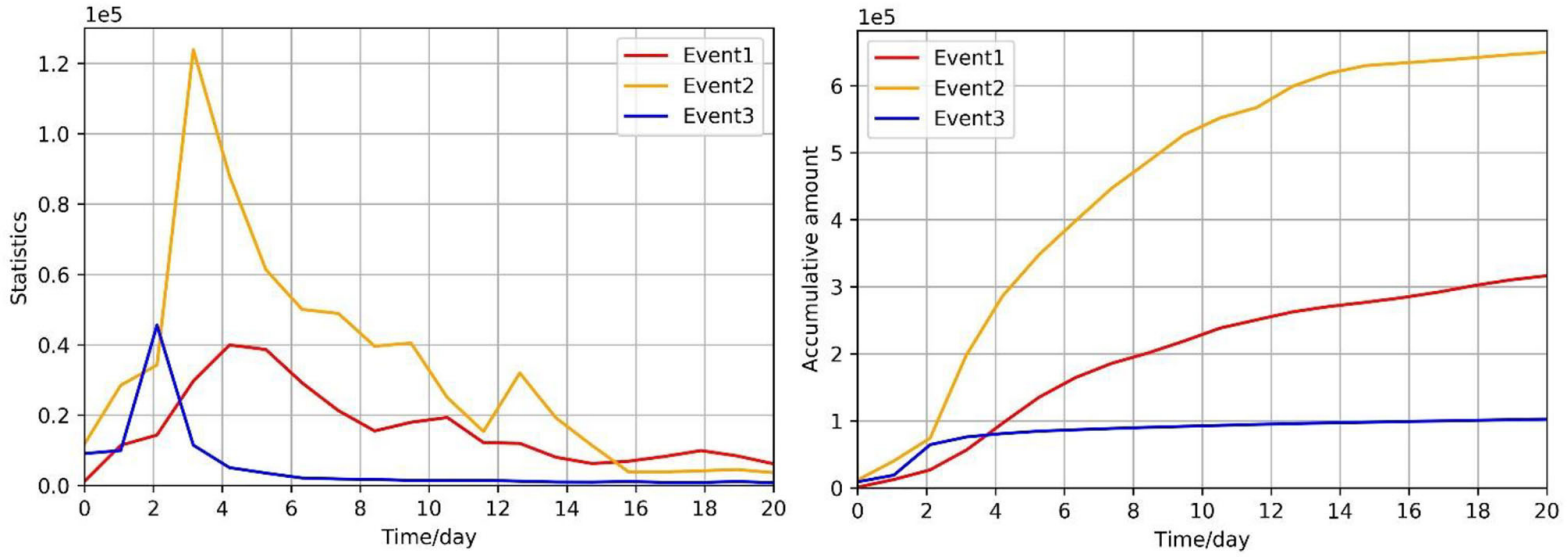
The amount of information on three network public opinion events.

The trend of network public opinion propagation can be expressed by the number of comments, forum posts, and messages written on social networking platforms. Figure 1 shows the real data of the above information of three different public opinion events. At the same time, this information can be represented in different moods and the curve of the amount of emotional information is also similar to the curve in Figure 1. This article regards the accumulation value of emotional information as a continuous variable that monotonously increases with time and describes the curve with the logistic model. The formula is as follows:


(1) $$\{\begin{array}{c} \frac{dx}{dt}\;=\;rx\left(1\;-\frac{x}{K}\right)\\ x\left(0\right)=x_{0} \end{array}$$

The natural growth rate of emotional information is *r* (*r* > 0). The initial value is *x*_0_. And the upper growth limit of the emotional information quantity is K.

### Transfer Model of Netizens' Emotions

In the processing of public opinion propagation, some netizens will influence other netizens' views on issues and change their emotions about these issues when they make comments and share their opinions, thus resulting in emotional transfer. To simulate this situation, we assume that *X*_1_, *X*_2_, *X*_3_, …, *Y* are different emotions and *x*_1_ = *x*_1_(*t*), *x*_2_ = *x*_2_(*t*), *x*_3_ = *x*_3_(*t*), …, *x*_*n*_ = *x*_*n*_(*t*), *y* = *y*(*t*) represent the emotional information quantity of them, respectively.

Emotion is the potential driving force of information dissemination in society (Stieglitz and Dang, 2013). When a focus event occurs in society, a large number of netizens' comments on the event are collected on the social platform, which implies the emotional tendency of netizens. The dissemination of information is essentially the dissemination of emotions. When the information on the social platform spreads from one netizen to another, the emotion attached to the information also spreads with the information. Because the transmission process of information is unidirectional and independent, we believe that the transmission of emotion is also unidirectional and independent. Therefore, we propose the following hypotheses to simplify the model:

Hypothesis 1: In the real scene, there are complex cases of mutual transfer between multiple emotions, but this model only considers the case of transfer from N emotions to a single emotion.

Hypothesis 2: The direction of emotion transfer is unidirectional, and there is no reverse transfer process.

Hypothesis 3: In the process of emotion transfer, each emotion is independent of the other and does not affect each other.

**Figure 2:**
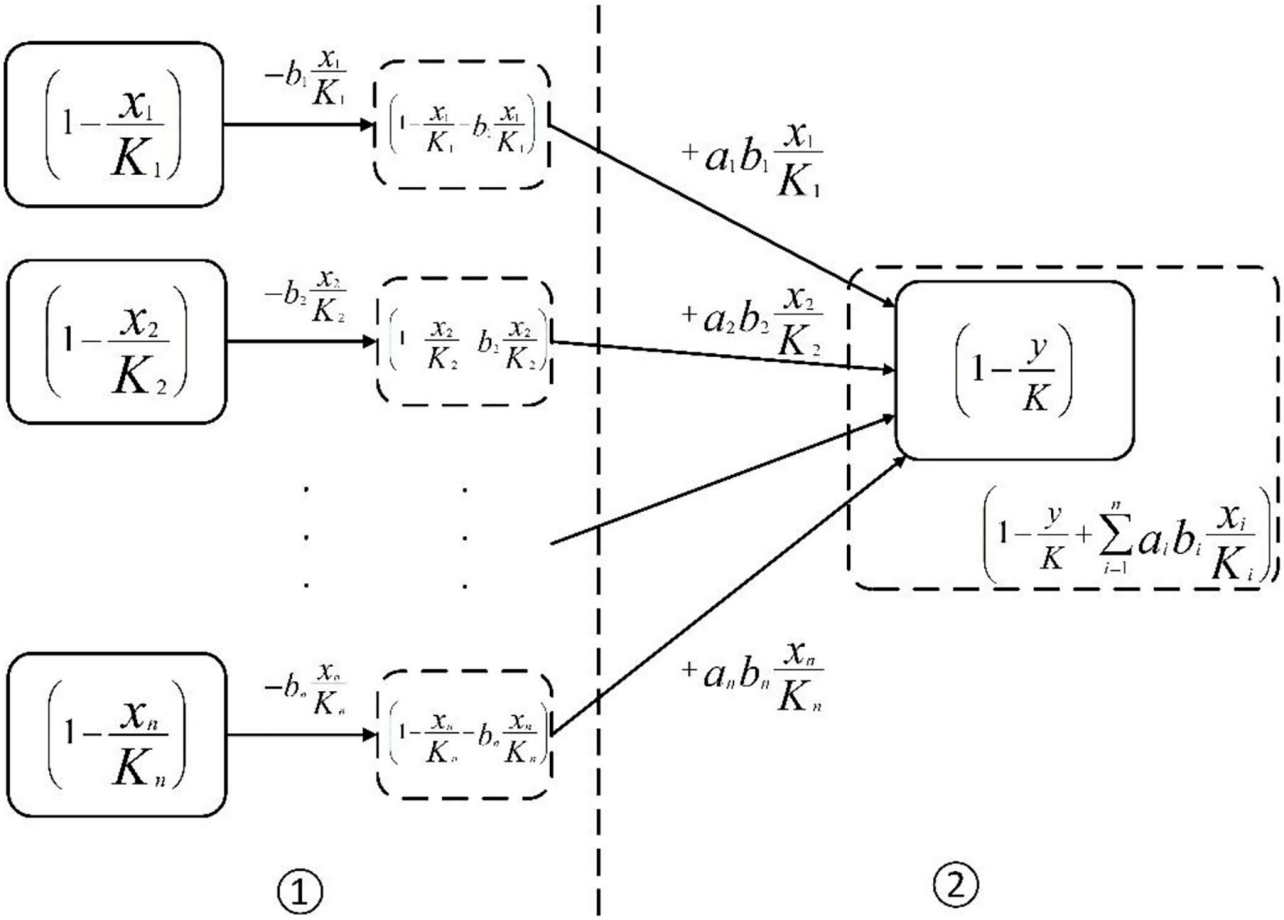
The transfer process of emotional information.

Based on the above hypotheses, there are *n* emotions (*X*_1_, *X*_2_, *X*_3_, …, *X*_*n*_) that transfer to one emotion (*Y*). First, the emotion information transferred from the *i*th emotion is $b_{i}\frac{x_{i}}{K_{i}}$, and *b*_*i*_(0< *b*_*i*_ < 1) is conversion proportion, which describes the proportion of the transferred emotion information amount to the original emotion information amount. After this process, the remaining space of *x*_*i*_ converts from $\left(1\;-\;\frac{x_{i}}{K_{i}}\;-\;b_{i}\frac{x_{i}}{K_{i}}\right)$ to $\left(1\;-\frac{x_{i}}{K_{i}}\right)$ (Figure 2, process ①). Then, we define transfer coefficient *a*_*i*_(0< *a*_*i*_ < 1). The emotional information $a_{i}b_{i}\frac{x_{i}}{K_{i}}$ which is a part of $b_{i}\frac{x_{i}}{K_{i}}$, transfers into *y*. The remaining space of *y* converts from $\left(1-\frac{y}{K}\right)$ to $\left(1-\frac{y}{K}+\overset{n}{\underset{i=1}{\sum }}a_{i}b_{i}\frac{x_{i}}{K_{i}}\right)$ (Figure 2, process ②). Based on the above two processes, the transfer model of netizens' emotions is constructed as:


(2) $$\{\begin{array}{c} \frac{dx_{1}}{dt}\;=\;r_{1}x_{1}\left(1-\frac{x_{1}}{K_{1}}-b_{1}\frac{x_{1}}{K_{1}}\right),x_{1}\left(0\right)\;=\;x_{10}\\ \frac{dx_{2}}{dt}=r_{2}x_{2}\left(1-\frac{x_{2}}{K_{2}}-b_{2}\frac{x_{2}}{K_{2}}\right),x_{2}\left(0\right)=x_{20}\\ .\\ .\\ .\\ \frac{dx_{n}}{dt}=r_{n}x_{n}\left(1-\frac{x_{n}}{K_{n}}-b_{n}\frac{x_{n}}{K_{n}}\right),x_{n}\left(0\right)=x_{n0}\\ \frac{dy}{dt}=ry\left(1-\frac{y}{K}+\sum_{i=1}^{n}a_{i}b_{i}\frac{x_{i}}{K_{i}}\right),y\left(0\right)=y_{0} \end{array}$$

where the initial values are *x*_10_,*x*_20_,*x*_30_,…,*x*_*n*0_,*y*_0_, the natural growth rates of emotional information are *r*_1_,*r*_2_,*r*_3_,…,*r*_*n*_,*r*, and the upper growth limits of the emotional information quantity are *K*_1_,*K*_2_,*K*_3_,…,*K*_*n*_,*K* (Table 1).

**Table 1:** Parameter meaning.

**Parameter**	**Meaning**
*t*	Time
*K*_*i*_	The upper growth limit of *x*_*i*_
*K*	The upper growth limit of *y*
*r*_*i*_	The intrinsic growth rate of *x*_*i*_
*r*	The intrinsic growth rate of *y*
*a*_*i*_	Transfer coefficient
*b*_*i*_	Conversion proportion

This model has no analytic solutions, but the stability of this model can be studied by calculating its equilibrium points. Set $\frac{dx_{1}}{dt}=\frac{dx_{2}}{dt}=\frac{dx_{3}}{dt}=...=\frac{dx_{n}}{dt}=\frac{dy}{dt}=0$, get equilibrium points. Considering *x*_1_, *x*_2_, *x*_3_, …, *x*_*n*_, *y* are continuous variables that are non-zero and monotonously increase with time, this model has the one and the only one non-zero equilibrium point $P\left(\frac{K_{1}}{{1+b}_{1}},\frac{K_{2}}{{1+b}_{2}},\frac{K_{3}}{{1+b}_{3}},...,\frac{K_{n}}{{1+b}_{n}},K+K\overset{n}{\underset{i=1}{\sum }}\frac{a_{i}b_{i}}{1+b_{i}}\;\right)$.

### Parameters Estimation

The parameters to estimate in this model include *K*_*i*_, *K*, *r*_*i*_, *r*, *a*_*i*_, and *b*_*i*_. We estimate the parameters by constructing difference equations and multiple linear regression. From Equation (2), we can get:


(3) $$\{\begin{array}{c} \frac{dx_{1}}{dt}=r_{1}x_{1}-\frac{r_{1}+r_{1}b_{1}}{K_{1}}x_{1}^{2}\\ \frac{dx_{2}}{dt}=r_{2}x_{2}-\frac{r_{2}+r_{2}b_{2}}{K_{2}}x_{2}^{2}\\ .\\ .\\ .\\ \frac{dx_{n}}{dt}=r_{n}x_{n}-\frac{r_{n}+r_{n}b_{n}}{K_{n}}x_{n}^{2}\\ \frac{dy}{dt}=ry-\frac{r}{K}y^{2}+r\sum_{i=1}^{n}a_{i}b_{i}\frac{x_{i}y}{K_{i}} \end{array}$$

According to the definition of differential and difference in mathematics, Δ*t* = *dt*, Δ*x*_*n*_ = *dx*_*n*_ + *O*(Δ*t*),*y* = *dy* + *O*(Δ*t*). When the unit time is taken (i.e., *t* = *dt* = 1), the differential equation model is constructed as:


(4) $$\{\begin{array}{c} \Delta x_{1}\left(k\right)=r_{1}x_{1}\left(k\right)-\frac{r_{1}+r_{1}b_{1}}{K_{1}}x_{1}^{2}\left(k\right)\\ \Delta x_{2}\left(k\right)=r_{2}x_{2}\left(k\right)-\frac{r_{2}+r_{2}b_{2}}{K_{2}}x_{2}^{2}\left(k\right)\\ .\\ .\\ .\\ \Delta x_{n}\left(k\right)=r_{n}x_{n}\left(k\right)-\frac{r_{n}+r_{n}b_{n}}{K_{n}}x_{n}^{2}\left(k\right)\\ \Delta y\left(k\right)=ry\left(k\right)-\frac{r}{K}y^{2}\left(k\right)+r\sum_{i=1}^{n}a_{i}b_{i}\frac{x_{i}\left(k\right)y\left(k\right)}{K_{i}} \end{array}$$

where Δ*x*_*i*_(*k*) = *x*_*i*_(*k*) − *x*_*i*_(*k* − 1) and Δ*y*(*k*) = *y*(*k*) − *y*(*k* − 1)(*k* = 1, 2, 3…, *i* = 1, 2, 3, …, *n*). The values of *x*_*i*_(*k*) and *y*(*k*) can be obtained by collecting real-time data on online public opinion. We can see that there is a linear relationship between the left side of the equations and the variables on the right side of the equations in Equation (3). Therefore, all parameters can be calculated using multiple linear regression analysis with real-time data as input.

## Simulation

**Figure 3:**
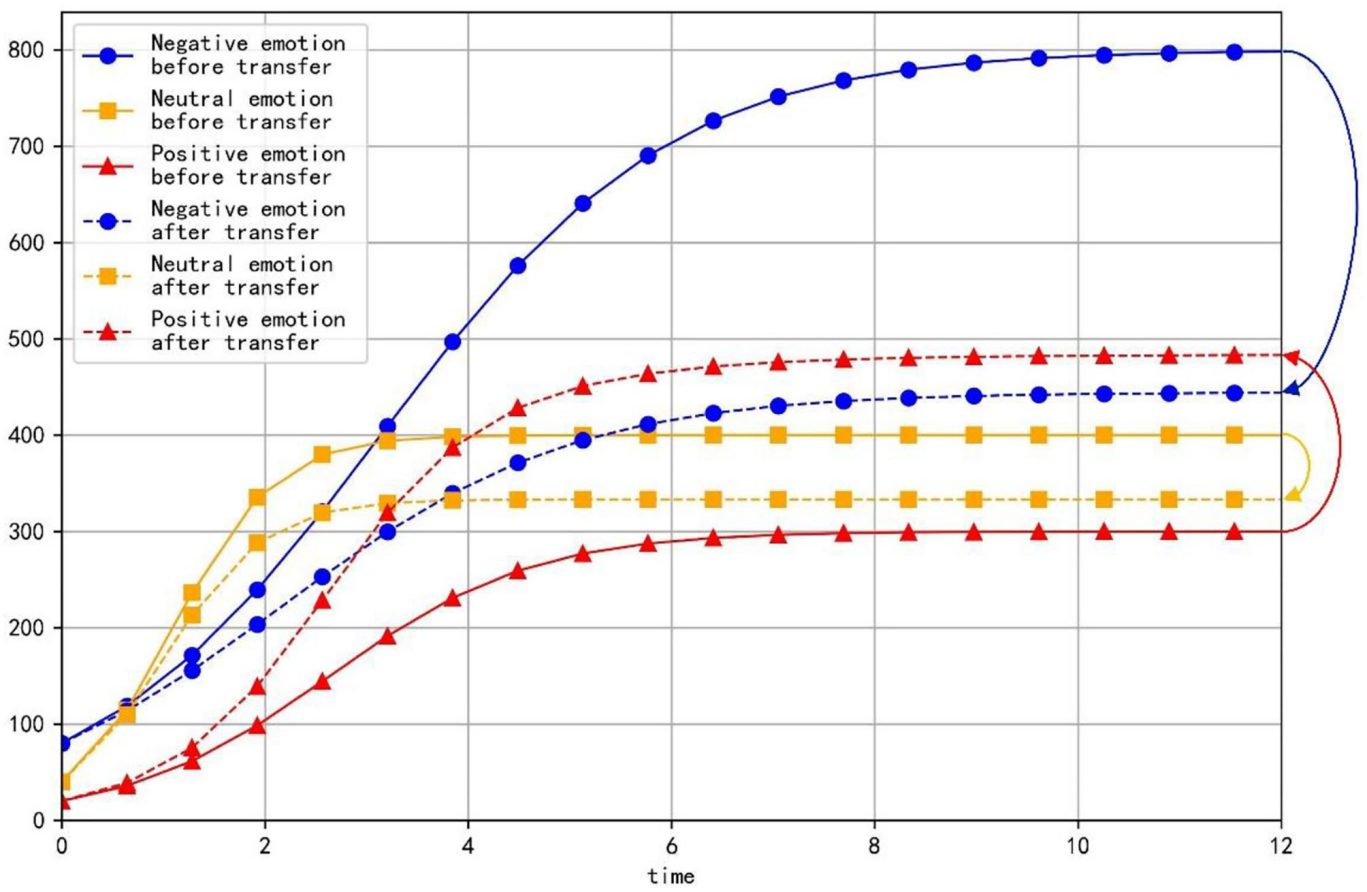
Comparison of emotional information before and after transfer.

Some major public crisis events often attract the attention of netizens on social networks, which causes netizens to have negative emotions such as anger and form a negative emotional scenario. In this case, relevant management departments need to adopt some guiding strategies to guide netizens to make positive comments and reduce negative words, so that neutral and negative emotions in the emotional scenario can be transferred to positive emotions (Figure 3). Under the effect of the guiding strategy, neutral emotion and negative emotion will decrease by a certain proportion. This proportion is represented by *b*_*i*_, which represents the guiding strength of guiding strategy. At the same time, due to the influence of various factors, only part of the reduced neutral and negative emotions will be transformed into positive emotions. This proportion is represented by *a*_*i*_, which represents the guiding efficiency of the guidance strategy.

This study simulates the influence of transfer coefficient and conversion proportion on transfer of emotions in the above scenario. The three variables *x*_1_(*t*), *x*_2_(*t*), and *y*(*t*) are defined to represent the amount of negative, neutral, and positive emotion information, respectively. The mathematical model is as follows:


(5) $$\{\begin{array}{c} \frac{dx_{1}}{dt}=r_{1}x_{1}\left(1-\frac{x_{1}}{K_{1}}-b_{1}\frac{x_{1}}{K_{1}}\right),x_{1}\left(0\right)=x_{10}\\ \frac{dx_{2}}{dt}=r_{2}x_{2}\left(1-\frac{x_{2}}{K_{2}}-b_{2}\frac{x_{2}}{K_{2}}\right),x_{2}\left(0\right)=x_{20}\\ \frac{dy}{dt}=ry\left(1-\frac{y}{K}+a_{1}b_{1}\frac{x_{1}}{K_{1}}+a_{2}b_{2}\frac{x_{2}}{K_{2}}\right),y\left(0\right)=y_{0} \end{array}$$

Three factors are studied in the simulation: (1) The influence of the transfer coefficient is studied when the value of conversion proportion is fixed. (2) The influence of conversion proportion is studied when the value of the transfer coefficient is fixed. (3) The influence of transfer coefficient and conversion proportion on the upper growth limit of positive emotion information is studied by setting different value combinations.

Considering the negative emotional scenario and the control effect of management departments, this study sets a small growth rate for negative emotional information, but a large upper growth limit. The specific parameters' information is shown in Table 2.

**Table 2:** Parameter values.

**Variable**	**The initial value**	**The intrinsic growth rate**	**The upper growth limit**
*x*_1_	*x*_10_ = 80	*r*_1_ = 0.7	*K*_1_ = 800
*x*_2_	*x*_20_ = 40	*r*_2_ = 2	*K*_2_ = 400
*y*	*y* = 20	*r* = 1	*K* = 300

### Controlling the Conversion Proportion

**Figure 4:**
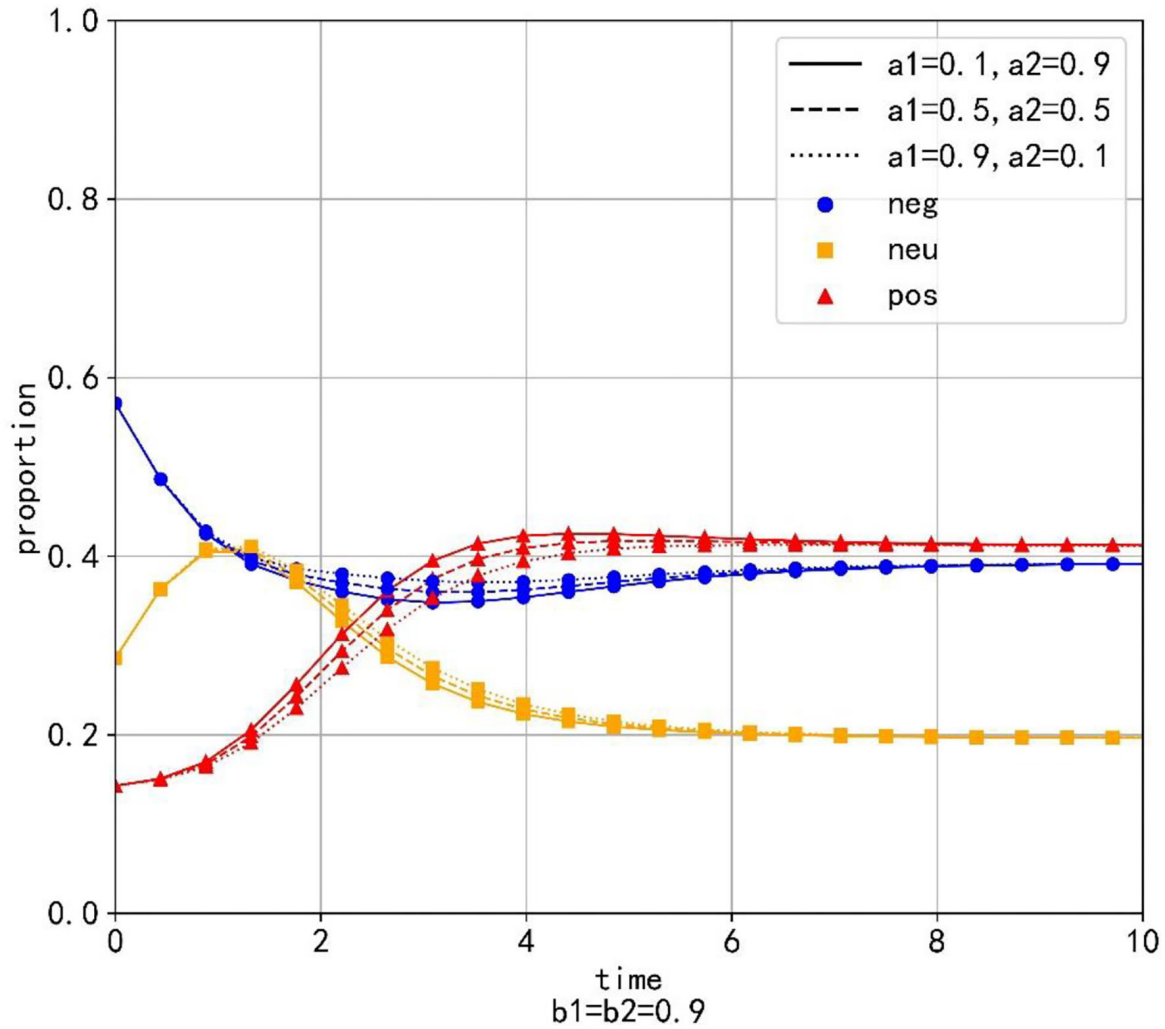
The evolution trend of the proportion of three emotions under the condition of changing the transfer coefficient.

Setting *b*_1_ = *b*_2_ = 0.9 and changing the values of *a*_1_ and *a*_2_, which are *a*_1_ = 0.1, *a*_1_ = 0.2, …, *a*_1_ = 0.9 and *a*_2_ = 0.9, *a*_2_ = 0.8, …, *a*_2_ = 0.1. Table 3 shows the values and proportions of the three emotions at the equilibrium point under different parameter values and Figure 4 shows the evolution trend of the proportions of the three emotions when *a*_1_ equals 0.1, 0.5, and 0.9, respectively.

**Table 3:** Simulation results under the condition of controlling the conversion proportion.

**Transfer coefficient**		**Conversion proportion**		**Positive emotion**		**Neutral emotion**		**Negative emotion**	
**a_1_**	**a_2_**	**b_1_**	**b_2_**	**Value**	**Proportion**	**Value**	**Proportion**	**Value**	**Proportion**
0.1	0.9	0.9	0.9	442.102	0.4117819	210.526	0.1960880	421.003	0.3921300
0.2	0.8	0.9	0.9	442.099	0.4117802	210.526	0.1960886	421.003	0.3921312
0.3	0.7	0.9	0.9	442.096	0.4117784	210.526	0.1960892	421.003	0.3921324
0.4	0.6	0.9	0.9	442.093	0.4117767	210.526	0.1960898	421.003	0.3921335
0.5	0.5	0.9	0.9	442.089	0.4117750	210.526	0.1960904	421.003	0.3921347
0.6	0.4	0.9	0.9	442.086	0.4117732	210.526	0.1960909	421.003	0.3921358
0.7	0.3	0.9	0.9	442.083	0.4117715	210.526	0.1960915	421.003	0.3921370
0.8	0.2	0.9	0.9	442.080	0.4117697	210.526	0.1960921	421.003	0.3921382
0.9	0.1	0.9	0.9	442.077	0.4117680	210.526	0.1960927	421.003	0.3921393

As is shown in Table 3, when the equilibrium point is reached, the proportion of positive emotion is higher than that of neutral emotion and negative emotion with any parameter values. Besides, with the increase of *a*_1_, the value and proportion of positive emotion decrease gradually, while the proportions of neutral emotion and negative emotion increase gradually at the equilibrium point. It can be seen that neutral emotion has a larger intrinsic growth rate than the negative emotion. Therefore, under the condition of constant conversion proportion, the larger *a*_2_ is, the more emotional information is transferred into positive emotion and the value and proportion of the positive emotion will be larger at the equilibrium point.

As is shown in Figure 4, different values of the transfer coefficients have no significant effect on the evolution trend of the three emotions. Because neutral emotion has a large intrinsic growth rate, the proportion of neutral emotion does not decrease immediately when emotion transfers out, but displays the tendency to rise at beginning and decline later. On the contrary, the intrinsic growth rate of negative emotion is small, so its proportion decreases immediately when the emotion transfer occurs. At the same time, with the decrease of *a*_2_, the proportion of positive emotion decreases, but the proportions of neutral emotion and negative emotion increase, which indicates that in the evolution process of emotion evolution, the emotion transferred out with a higher growth rate is the main factor determining the proportion of emotion transferred in.

As can be seen from the above simulation results, when relevant departments implement public opinion guidance, the guiding strength of guidance strategy is the primary factor determining the final guidance effect, while the efficiency of guidance strategy is only a secondary factor. Therefore, relevant departments should invest enough force to make neutral emotion and negative emotion transfer out in a large proportion as far as possible. At the same time, when certain guiding strength is applied, we should first consider improving the guiding efficiency of the emotion with a higher growth rate, so that the transferring part of the emotion is transferred to positive emotion in a larger proportion, which will be more conducive to obtaining good guiding effect.

### Controlling the Transfer Coefficient

**Figure 5:**
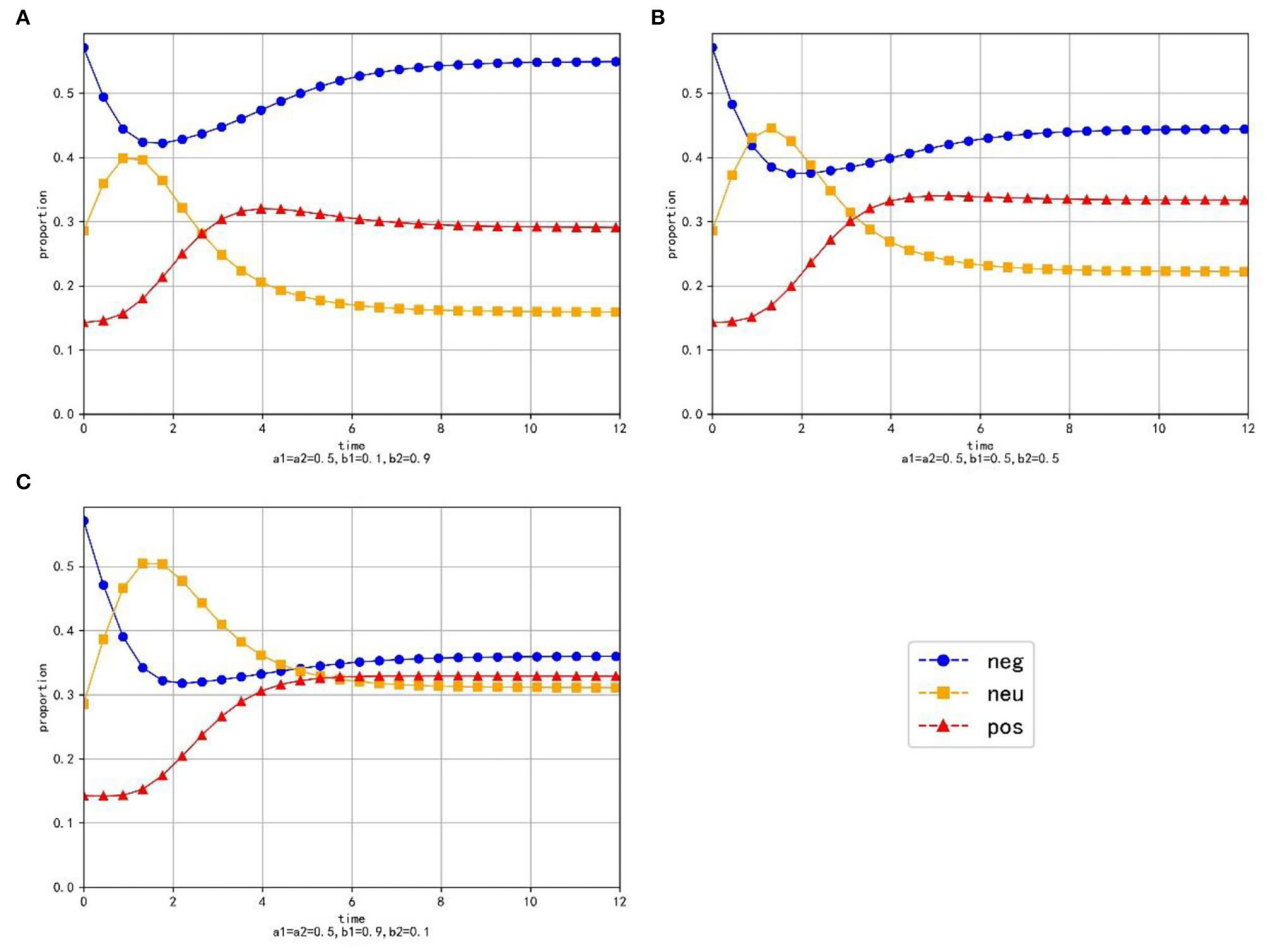
The evolution trend of the proportion of three emotions under the condition of changing the conversion proportion. **(A)** When *a*_1_ = *a*_2_ = 0.5, *b*_1_ = 0.2 and *b*_2_ = 0.9. **(B)** When *a*_1_ = *a*_2_ = 0.5, *b*_1_ = 0.5 and *b*_2_ = 0.5. **(C)** When *a*_1_ = *a*_2_ = 0.5, *b*_1_ = 0.9 and *b*_2_ = 0.1.

Setting *a*_1_ = *a*_2_ = 0.5 and changing the values of *b*_1_ and *b*_2_, which are *b*_1_ = 0.1, *b*_1_ = 0.2, …, *b*_1_ = 0.9 and *b*_2_ = 0.9, *b*_2_ = 0.8, …, *b*_2_ = 0.1. Table 4 shows the values and proportions of the three emotions at the equilibrium point under different parameter values and Figure 5 shows the evolution trend of the proportions of the three emotions when *b*_1_ equals 0.1, 0.5, and 0.9, respectively.

**Table 4:** Simulation results under the condition of controlling the transfer coefficient.

**Transfer coefficient**		**Conversion proportion**		**Positive emotion**		**Neutral emotion**		**Negative emotion**	
**a_1_**	**a_2_**	**b_1_**	**b_2_**	**Value**	**Proportion**	**Value**	**Proportion**	**Value**	**Proportion**
0.5	0.5	0.1	0.9	384.682	0.2909148	210.526	0.1592099	727.111	0.5498753
0.5	0.5	0.2	0.8	391.656	0.3058831	222.222	0.1735555	666.532	0.5205614
0.5	0.5	0.3	0.7	396.367	0.3178734	235.294	0.1886985	615.271	0.4934281
0.5	0.5	0.4	0.6	399.092	0.3270109	250.000	0.2048469	571.332	0.4681422
0.5	0.5	0.5	0.5	399.984	0.3333473	266.667	0.2222407	533.250	0.4444120
0.5	0.5	0.6	0.4	399.090	0.3368610	285.714	0.2411636	499.928	0.4219754
0.5	0.5	0.7	0.3	396.362	0.3374504	307.692	0.2619595	470.525	0.4005901
0.5	0.5	0.8	0.2	391.649	0.3349226	333.333	0.2850536	444.389	0.3800238
0.5	0.5	0.9	0.1	384.671	0.3289723	363.636	0.3109837	421.003	0.3600441

As is shown in Table 4, at the equilibrium point, the proportions and values of neutral emotion and negative emotion are inversely proportional to their conversion proportions, respectively. When *b*_1_ increases and *b*_2_ decreases, the value and proportion of neutral emotion gradually increase, the value and proportion of negative emotion gradually decrease, while the value and proportion of positive emotion first increase then decrease (the evolution law of positive emotion is specifically discussed in Section The Influence of Transfer Coefficient and Conversion Proportion on Positive Emotion).

As is shown in Figure 5, the proportion of neutral emotion has a trend of rising first and falling with time. With the decrease of *b*_2_, the peak value of negative emotion curve increases obviously and the rate of decline gradually slows down. Since the growth rate of negative emotion is small, the proportion of negative emotion decreases at the beginning, however, as time goes by, the information transferred from negative emotion decreases gradually and the negative information that grows naturally is enough to make up for the amount of information transferred out, then the proportion of negative emotion increases in the later period. With the increase of *b*_1_, the minimum value of the curve of negative emotion becomes smaller and the ascending process of the curve gradually becomes less obvious. The proportion of positive emotion increases at the beginning, but in the later period, the emotional information transferred from negative emotion and neutral emotion decreases, which leads to a decrease in the proportion of positive emotion. With the increase of *b*_1_, the downward trend of positive emotion gradually becomes gentle in the later period.

The simulation results show that when the guiding efficiency of the guiding strategy is certain, the change of the guiding strength greatly affects the final guiding effect of public opinion, which further confirms the view that the guiding force is the primary consideration for relevant departments to implement public opinion guidance. In addition, for the emotion with a large growth rate, its proportion cannot be inhibited in the early stage under a certain guiding strength. This shows once again that the emotion with a large growth rate is the focus of guidance, and it needs to be guided vigorously for a long time to effectively reduce its proportion and get a better guidance effect.

### The Influence of Transfer Coefficient and Conversion Proportion on Positive Emotion

As can be seen from Equation (4), the value of *y* at the equilibrium point is related to parameters *a*_1_, *a*_2_, *b*_1_, and *b*_2_, and the simulation results in Table 4 show that, when the transfer coefficient is fixed, the value of *y* at the equilibrium point increases first and then decreases with the increase of *b*_1_ and the decrease of *b*_2_, which is not directly or inversely proportional to the value of conversion proportion. In simulations 3.1 and 3.2, the influence of *a*_1_ and *a*_2_ (or *b*_1_ and *b*_2_) on the value of positive emotion at the equilibrium point was explored only when the values of *b*_1_ and *b*_2_ (or *a*_1_ and *a*_2_) were fixed, which cannot comprehensively display the change trend of positive emotion value at the equilibrium point when *a*_1_, *a*_2_, *b*_1_, and *b*_2_ change continuously at the same time. Therefore, this study designed four groups of simulation experiments, corresponding to four different scenarios of neutral and negative emotion transfer to positive emotion. Calculating the value of positive emotion at the equilibrium point under different values of each parameter, the values of parameters *a*_1_, *a*_2_, *b*_1_, and *b*_2_ in each simulation are as follows:

Simulation 1: Set *a*_2_ = *a*_1_, *b*_2_ = *b*_1_, where *a*_1_ values 0.1, 0.2, 0.3, …, 0.9 in turn. For each value of *a*_1_, *b*_1_ values 0.1, 0.2, 0.3, …, 0.9.

Simulation 2: Set *a*_2_ = *a*_1_, *b*_2_ = 1 − *b*_1_, where *a*_1_ values 0.1, 0.2, 0.3, …, 0.9 in turn. For each value of *a*_1_, *b*_1_ values 0.1, 0.2, 0.3, …, 0.9.

Simulation 3: Set *a*_2_ = 1 − *a*_1_, *b*_2_ = *b*_1_, where *a*_1_ values 0.1, 0.2, 0.3, …, 0.9 in turn. For each value of *a*_1_, *b*_1_ values 0.1, 0.2, 0.3, …, 0.9.

Simulation 4: Set *a*_2_ = 1 − *a*_1_, *b*_2_ = 1 − *b*_1_, where *a*_1_ values 0.1, 0.2, 0.3, …, 0.9 in turn. For each value of *a*_1_, *b*_1_ values 0.1, 0.2, 0.3, …, 0.9.

Taking the simulation 1 as an example, the values of *a*_1_, *a*_2_, *b*_1_ and *b*_2_ are as follows (Table 5):

**Table 5:** Parameter values in four simulation scenarios.

	**b_1_**	**b_2_**
**a**_**1**_ **= a**_**2**_ **= 0.1**	0.1 0.2 0.3	0.1 0.2 0.3
	0.4 0.5 0.6	0.4 0.5 0.6
	0.7 0.8 0.9	0.7 0.8 0.9
**a**_**1**_ **= a**_**2**_** = 0.2**	0.1 0.2 0.3	0.1 0.2 0.3
	0.4 0.5 0.6	0.4 0.5 0.6
	0.7 0.8 0.9	0.7 0.8 0.9
**a**_**1**_ **= a**_**2**_** = 0.9**	0.1 0.2 0.3	0.1 0.2 0.3
	0.4 0.5 0.6	0.4 0.5 0.6
	0.7 0.8 0.9	0.7 0.8 0.9

**Figure 6:**
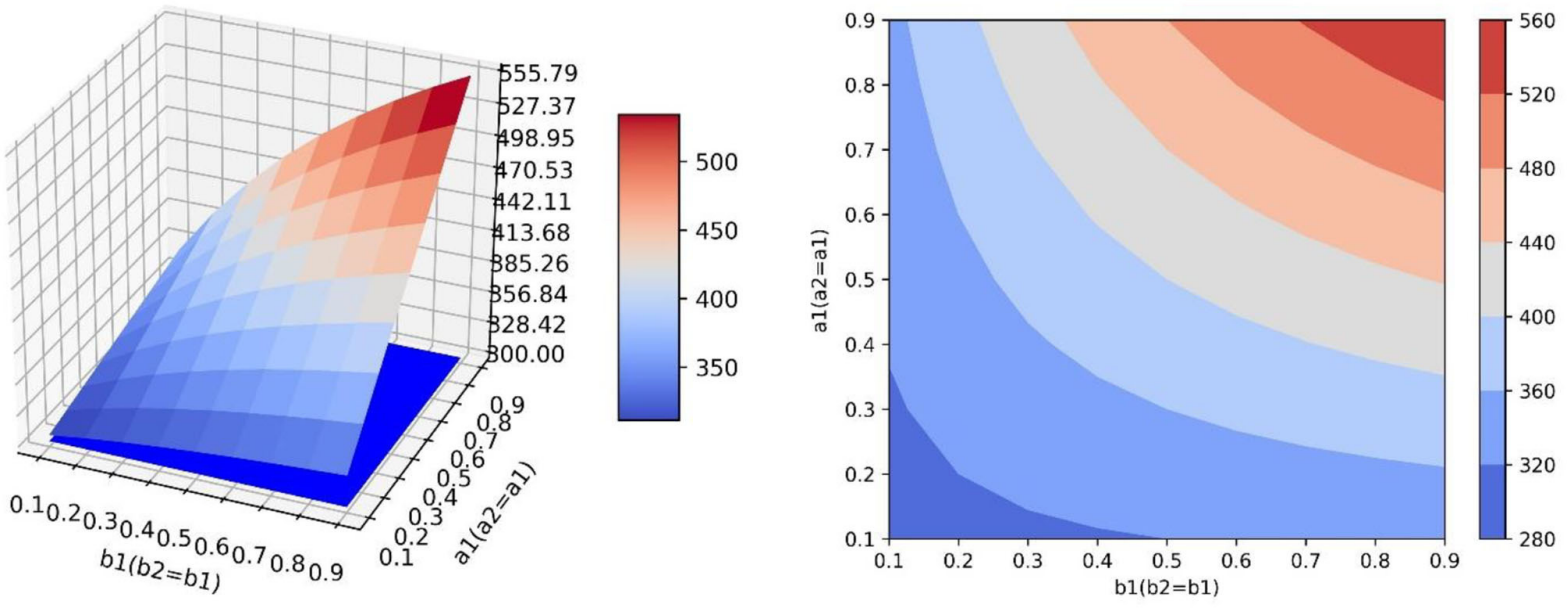
In the scene of simulation 1, the value of positive emotion at the equilibrium point: surface graph and surface projection.

In the scenario of simulation 1, it is assumed that relevant government departments have sufficient resources to implement guidance strategies and can efficiently guide both neutral emotion and negative emotion. Therefore, parameters *a*_1_, *a*_2_, *b*_1_, and *b*_2_ can increase synchronously. This scenario is the most ideal guiding scenario. When *a*_1_ = *a*_2_ = *b*_1_ = *b*_2_=0.9, the optimal guiding effect can be achieved, the value of positive emotion quantity *y* is 555.789, and the proportion of emotion quantity is 0.4680938 (Figure 6). It can be seen from the simulation results that the change rates of the surface along the direction *a*_1_ and *b*_1_ are similar, that is to say, the changes of *a* and *b* have similar effects on the changes of *y*. When *a*_1_ and *b*_1_ increase synchronously, it has great benefit to improving the guiding effect.

**Figure 7:**
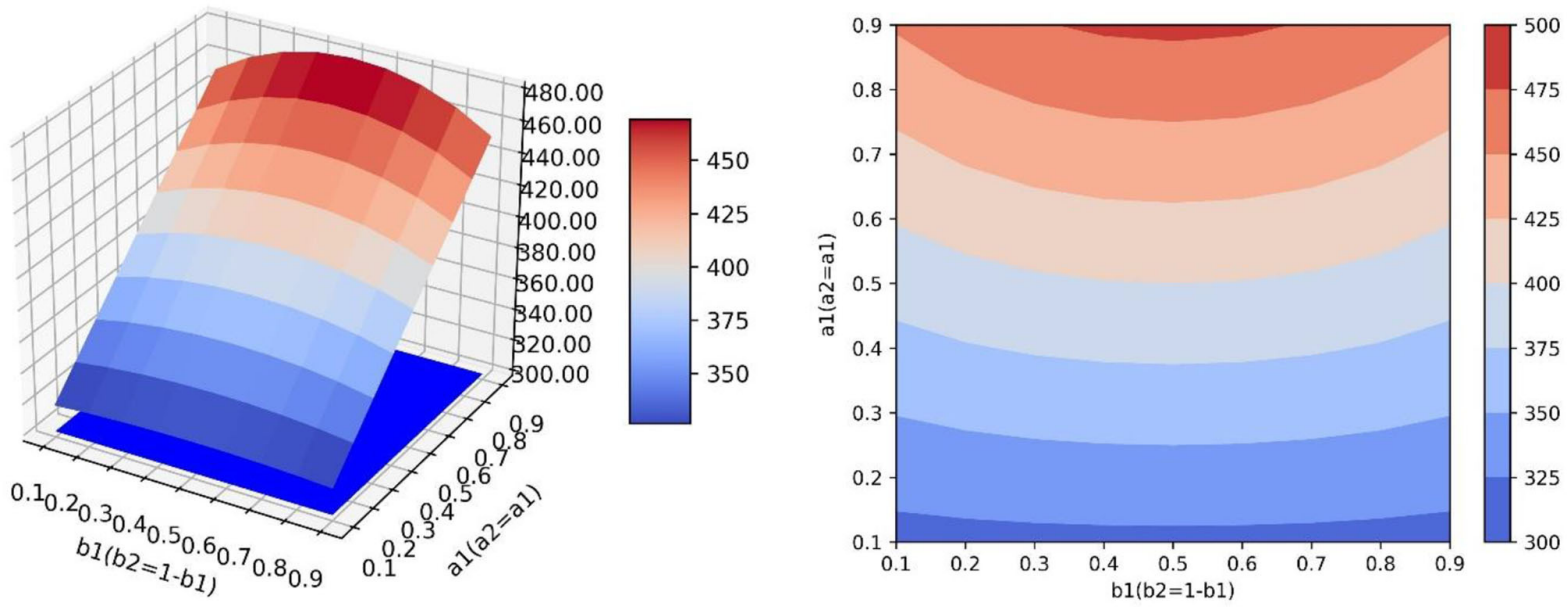
In the scene of simulation 2, the value of positive emotion at the equilibrium point: surface graph and surface projection.

**Figure 8:**
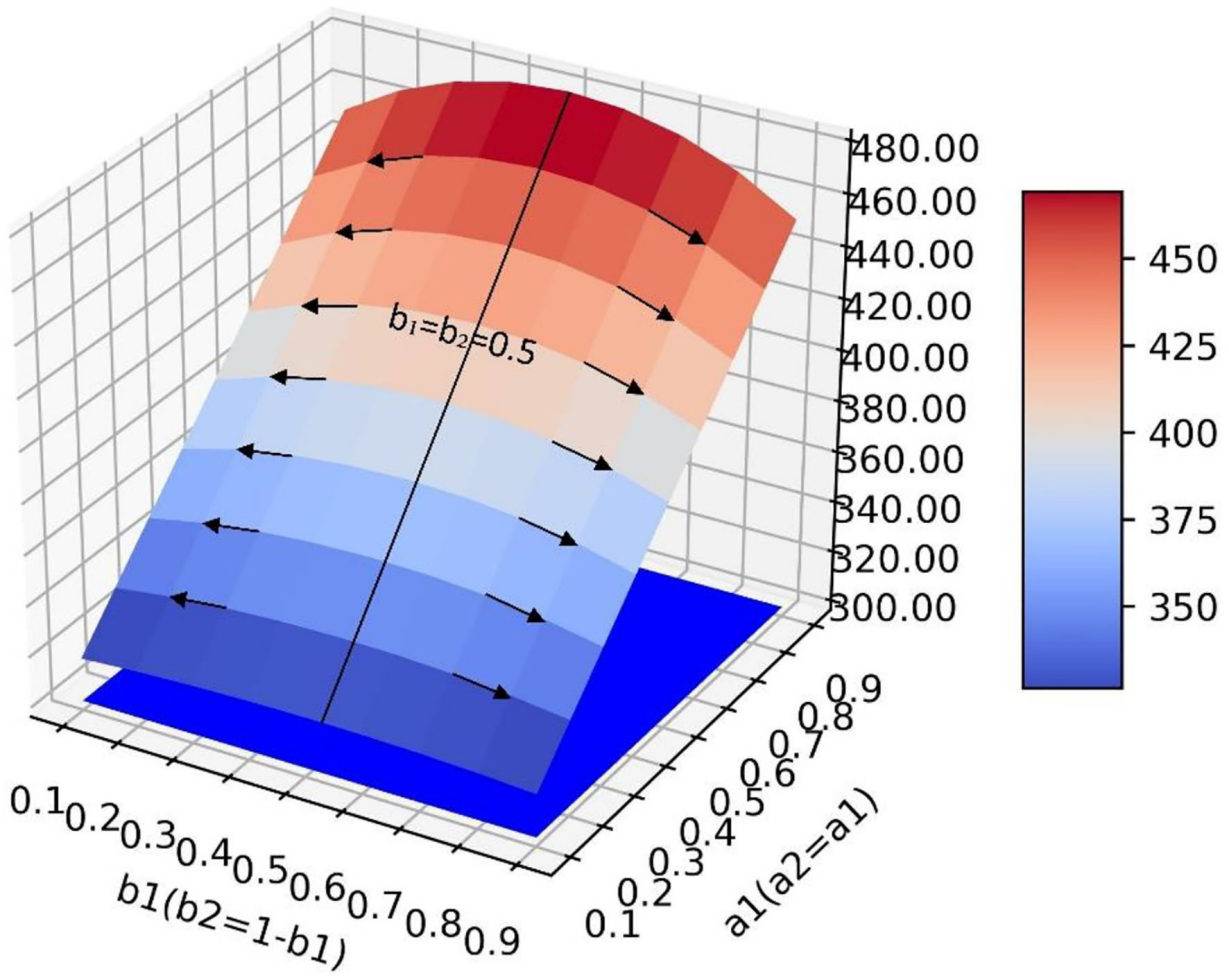
In the scene of simulation 2, the local optimal value of Y.

In the scenario of simulation 2, it is assumed that relevant government departments do not have sufficient resources to guide both neutral emotion and negative emotion. When one emotion is guided with great strength, the other emotion is guided with little strength. Therefore, for *b*_1_ and *b*_2_, when the value of one of them increases, the value of the other one decreases. However, in this scenario, the two emotions can be guided with high efficiency at the same time, so *a*_1_ and *a*_2_ can increase synchronously (Figure 7). It can be seen that since *a*_1_ and *a*_2_ can increase synchronously, the change rate of the surface along the *a*_1_ direction is larger. In this scenario, compared with increasing the value of *b*_1_, increasing the value of *a*_1_ is more beneficial for improving the guidance effect. By analyzing changes along the *b*_1_ axis, it can be seen that when *a*_1_ has a constant value and *b*_1_ = *b*_2_ = 0.5, the local optimal value of *y* can be obtained. At this point, the value of *y* will decrease regardless of increasing or decreasing the value of *b*_1_ (Figure 8). This is because when the value of one of *b*_1_ and *b*_2_ increases, the value of the other will decrease. Only when the value of the two parameters is considered comprehensively (the guiding strength of neutral emotion and negative emotion is considered comprehensively in the implementation of guidance), and the maximum value of *b*_2_ can be achieved, the better guidance effect can be achieved.

**Figure 9:**
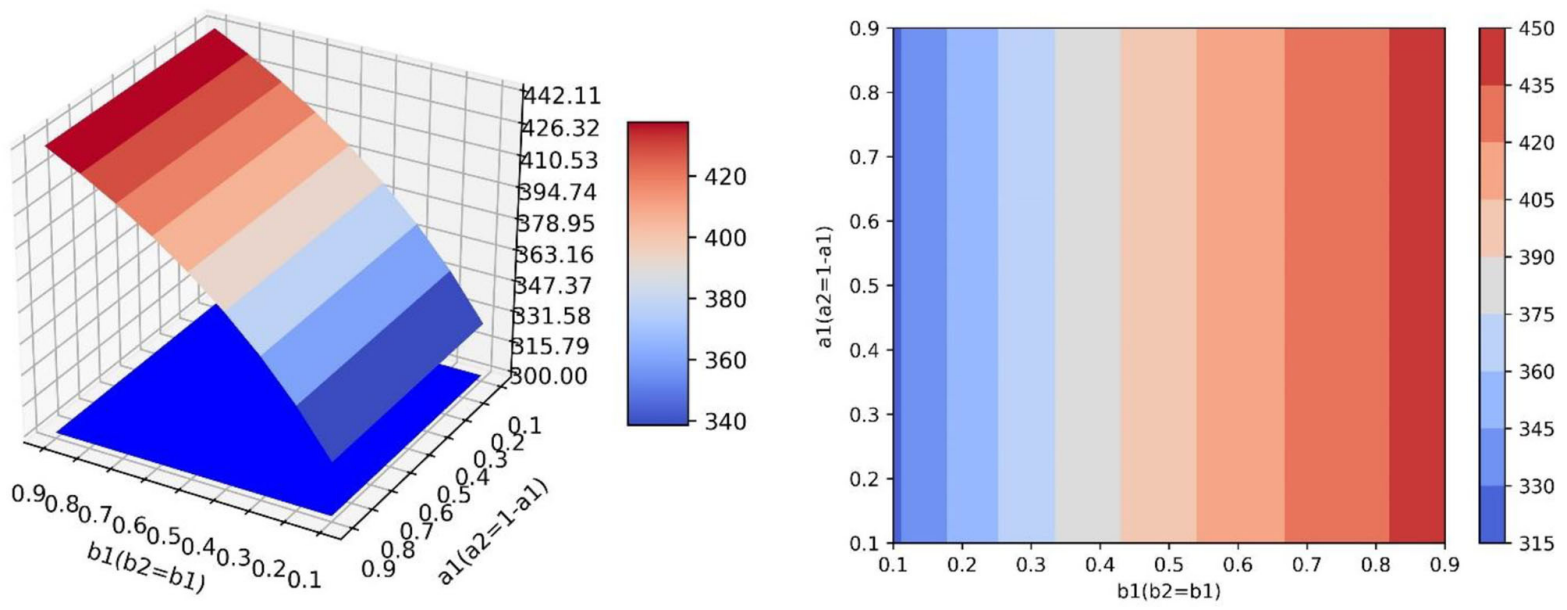
In the scene of simulation 3, the value of positive emotion at the equilibrium point: surface graph and surface projection.

In the scenario of simulation 3, it is assumed that relevant government departments have sufficient resources to guide both neutral and negative emotions, therefore, parameters *b*_1_ and *b*_2_ can increase synchronously. However, in this scenario, the guidance efficiency of the guidance strategy is not high, and it cannot have high guidance efficiency for two emotions at the same time, and the values of one of *a*_1_ and *a*_2_ show that one increases and the other decreases (Figure 9). This scenario is contrary to the scenario of simulation 2. Changing the value of *a*_1_ does not influence the improvement of the guidance effect, while increasing the value of *b*_1_ has great benefits on the improvement of the guidance effect.

**Figure 10:**
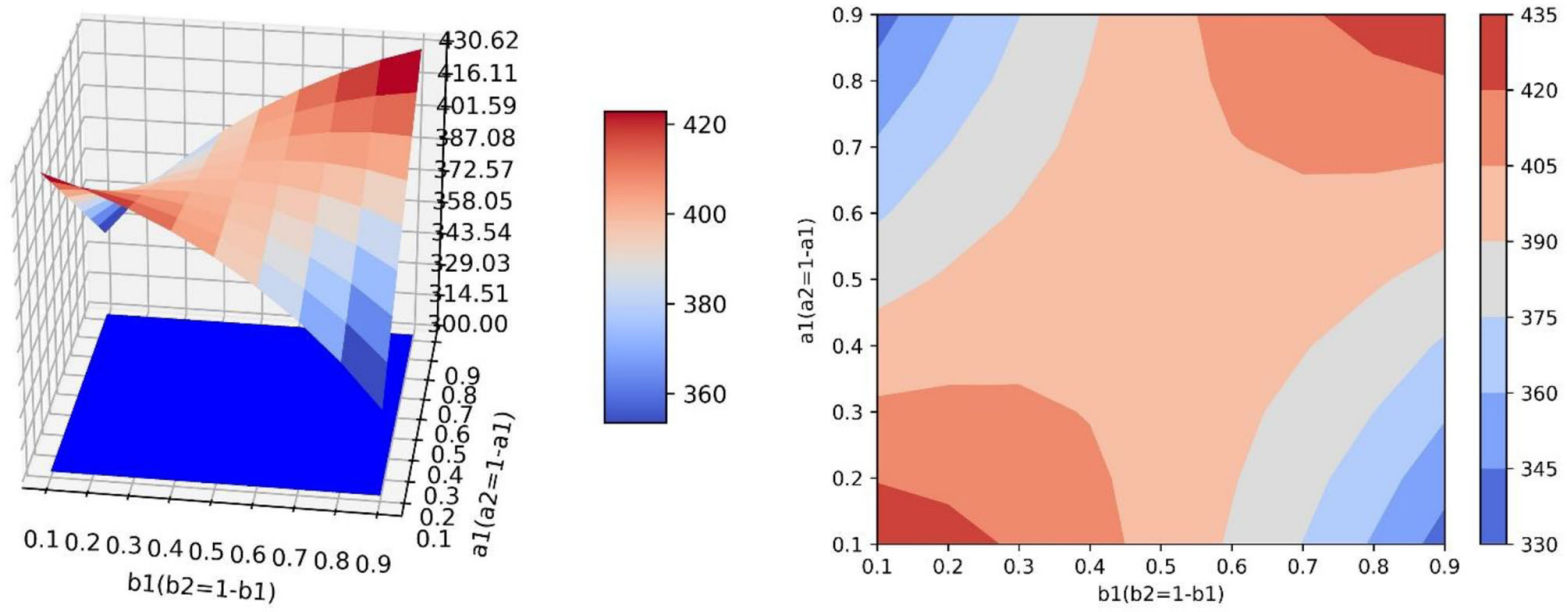
In the scene of simulation 4, the value of positive emotion at the equilibrium point: surface graph and surface projection.

**Figure 11:**
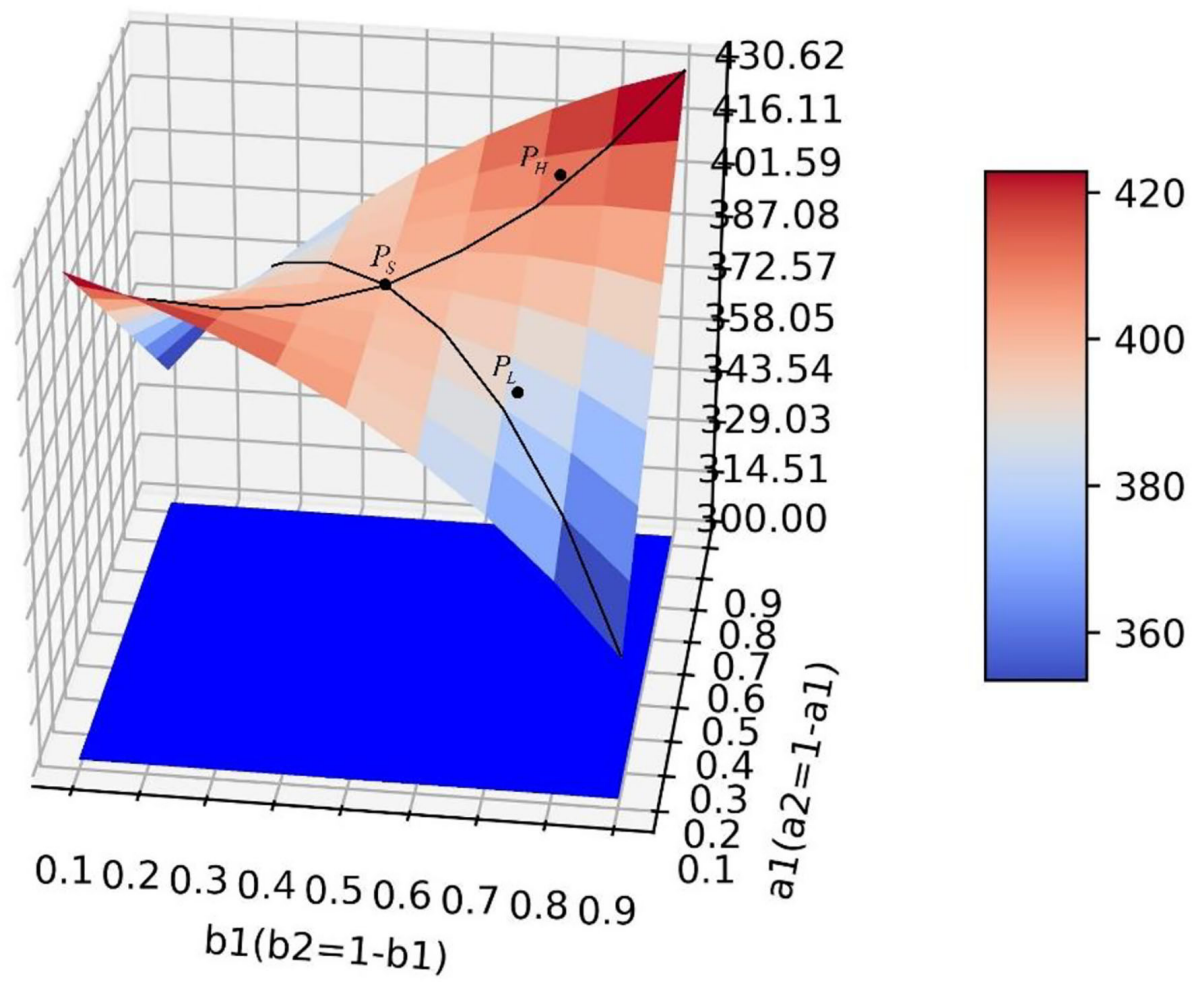
In the scene of simulation 4, the saddle point *P*_*S*_ on the surface.

In the scenario of simulation 4, it is assumed that relevant government departments neither have sufficient resources to guide both neutral and negative emotions at the same time (parameters *b*_1_ and *b*_2_ cannot increase synchronously), nor can they have high guidance efficiency for both emotions at the same time (parameters *a*_1_ and *a*_2_ cannot increase synchronously) (Figure 10). In this scenario, there is a saddle point *P*_*S*_ on the surface, and the influence of the values of parameters on the value of *y* needs to be discussed separately. When the position of point *P*_*L*_ is lower than the saddle point, the value of *a*_1_ should be increased and the value of *b*_1_ should be decreased to improve the guiding effect. However, when the position of point *P*_*H*_ is lower than the saddle point, the values of *a*_1_ and *b*_1_ need to be increased simultaneously to improve the guiding effect (Figure 11).

It can be concluded from the above four simulation results that different guidance strategies need to be adopted in different scenarios to improve the guidance effect, which cannot be qualitatively generalized. Therefore, it is necessary to put forward a quantitative optimization method for guiding strategies in different scenarios to effectively deal with them. The next section presents the specific approach to this problem.

### Optimization Method of Guidance Strategy

**Figure 12:**
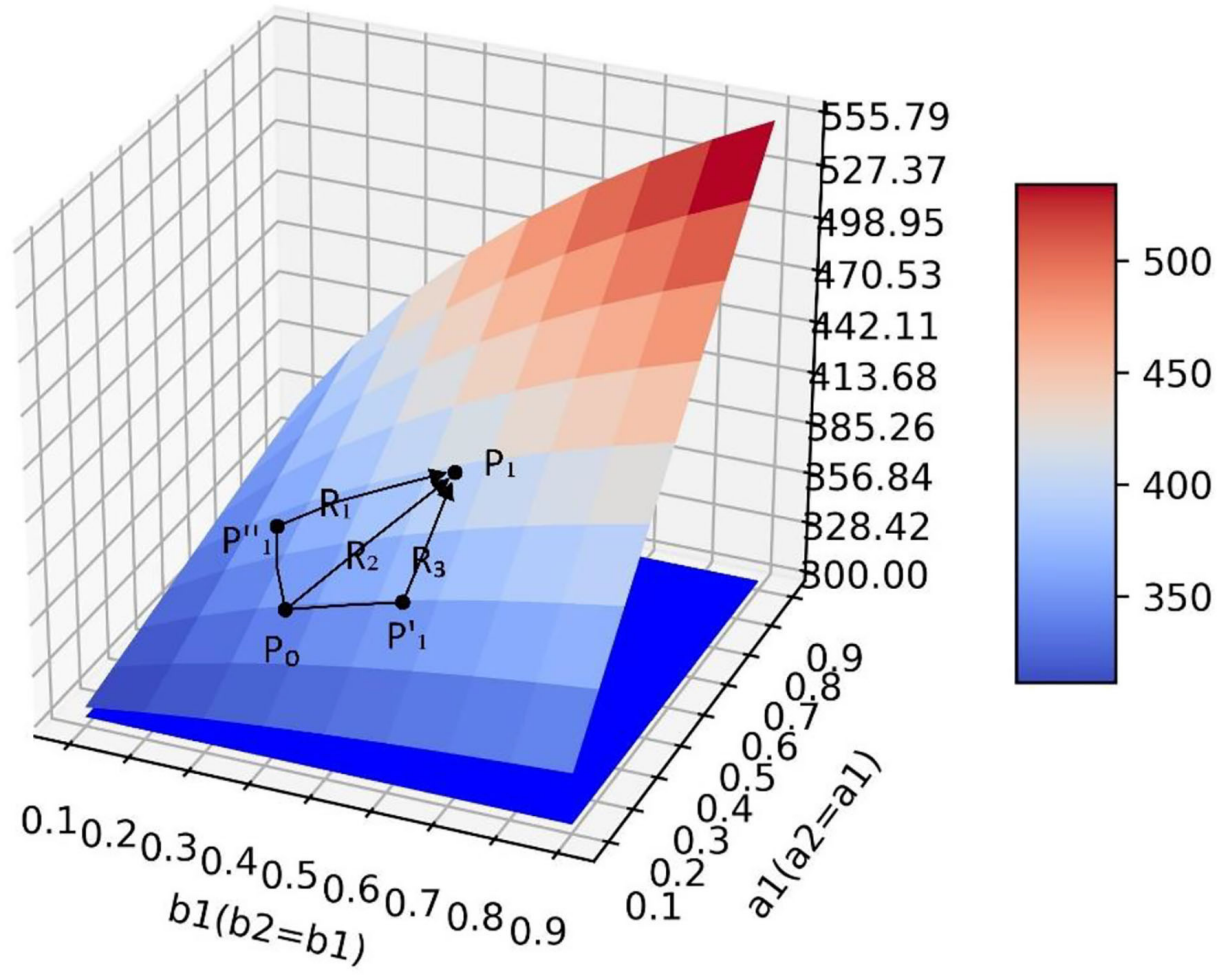
Different policy paths on the surface.

The analysis in Section The Influence of Transfer Coefficient and Conversion Proportion on Positive Emotion is only a qualitative analysis by observing simulation results. To quantitatively analyze how to take optimal steps to optimize the current guidance strategy in actual guidance scenarios, this section takes simulation 1 as an example and makes the following definitions (Figure 12): point *P*_0_ represents the guidance effect under the current guidance strategy, at this point, the quantity of positive emotional information *y* = *y*_*P*_0__, and $a_{1}=a_{1}^{<0>},a_{2}=a_{2}^{<0>},b_{1}=b_{1}^{<0>},b_{2}\;=\;b_{2}^{<0>}$. We define the values of the parameters as a guidance strategy, represented by a five-tuple:


$$\begin{array}{l} P_{0}\left(y_{P_{0}},a_{1}^{<0>},a_{2}^{<0>},b_{1}^{<0>},b_{2}^{<0>}\right) \end{array}$$

To achieve a more ideal guiding effect $P_{1}\left(y_{P_{1}},a_{1}^{<1>},a_{2}^{<1>},b_{1}^{<1>},b_{2}^{<1>}\right)$, it is necessary to adjust the parameter values so that the guidance policy $a_{1}=a_{1}^{<0>},a_{2}=a_{2}^{<0>},b_{1}=b_{1}^{<0>},b_{2}=b_{2}^{<0>}$ is adjusted to the better guidance policy $a_{1}=a_{1}^{<1>},a_{2}=a_{2}^{<1>},b_{1}=b_{1}^{<1>},b_{2}=b_{2}^{<1>}$. As shown in Figure 12, there are multiple paths from *P*_0_ to *P*_1_ on the surface. Paths *R*_1_, *R*_2_, and *R*_3_ are defined as policy paths which are the directions that each parameter should be adjusted under the current guidance policy. It can be seen that although the *P*_1_ point can be reached along different policy paths, the policy path *R*_2_ has a shorter path distance and can reach *P*_1_ point faster than the other two paths which need to go through the intermediate nodes $P_{1}^{{}^{\prime }}$ and $P_{1}^{{}^{\prime \prime }}$, and it takes less time in implementing the guidance strategy. Therefore, it is a better path.

Mathematically, there are infinite directional derivatives at any point on a smooth three-dimensional surface, and the value of the function increases fastest along the gradient direction. Therefore, the gradient direction is the optimal direction to select the strategic path under the current guidance strategy, and the problem of finding the optimal strategic path is transformed into the problem of finding the gradient direction. We can find the optimal strategy path by the gradient ascent method. Assuming *y* = *f*(*a*_1_, *b*_1_) is a binary function of *a*_1_ and *b*_1_, which has first-order continuous partial derivative. So for every point *P*(*a*_1_, *b*_1_), we get a vector $\frac{\partial y}{\partial a_{1}}\left(a_{1},b_{1}\right)\bar{i}+\frac{\partial y}{\partial b_{1}}\left(a_{1},b_{1}\right)\bar{j}$ and the gradient of function *f*(*a*_1_, *b*_1_) at point *P*(*a*_1_, *b*_1_) is ∇*f*(*a*_1_, *b*_1_). In order to find the gradient at any point on the surface, 81 sets of simulation data are used to fit the surface by the least square method. The surface equation in simulation 1 is $f\left(a_{1},b_{1}\right)\;=\;0.084a_{1}^{2}\;+\;281.0a_{1}b_{1}\;-\;95.18b_{1}^{2}\;+\;46.88a_{1}\;+\;95.55b_{1}\;+\;281.7$ and the gradient of the surface at any point is $\nabla f\left(a_{1},b_{1}\right)\;=\;\left(0.168a_{1}\;+\;281.0b_{1}\;+\;46.88\right)\bar{i}\;+\;\left(-190.36b_{1}\;+\;281.0a_{1}\;+\;95.55\right)\;\bar{j}$.

After obtaining the gradient equation, the optimal strategy path can be obtained by iterating Equation (5).


(6) $$\{\begin{array}{c} a_{1}^{<n+1>}=a_{1}^{<n>}+\gamma \left(0.168a_{1}^{<n>}+281.0b_{1}^{<n>}+46.88\right)\\ b_{1}^{<n+1>}=b_{1}^{<n>}+\gamma \left(-190.36b_{1}^{<n>}+281.0a_{1}^{<n>}+95.55\right) \end{array}$$

**Figure 13:**
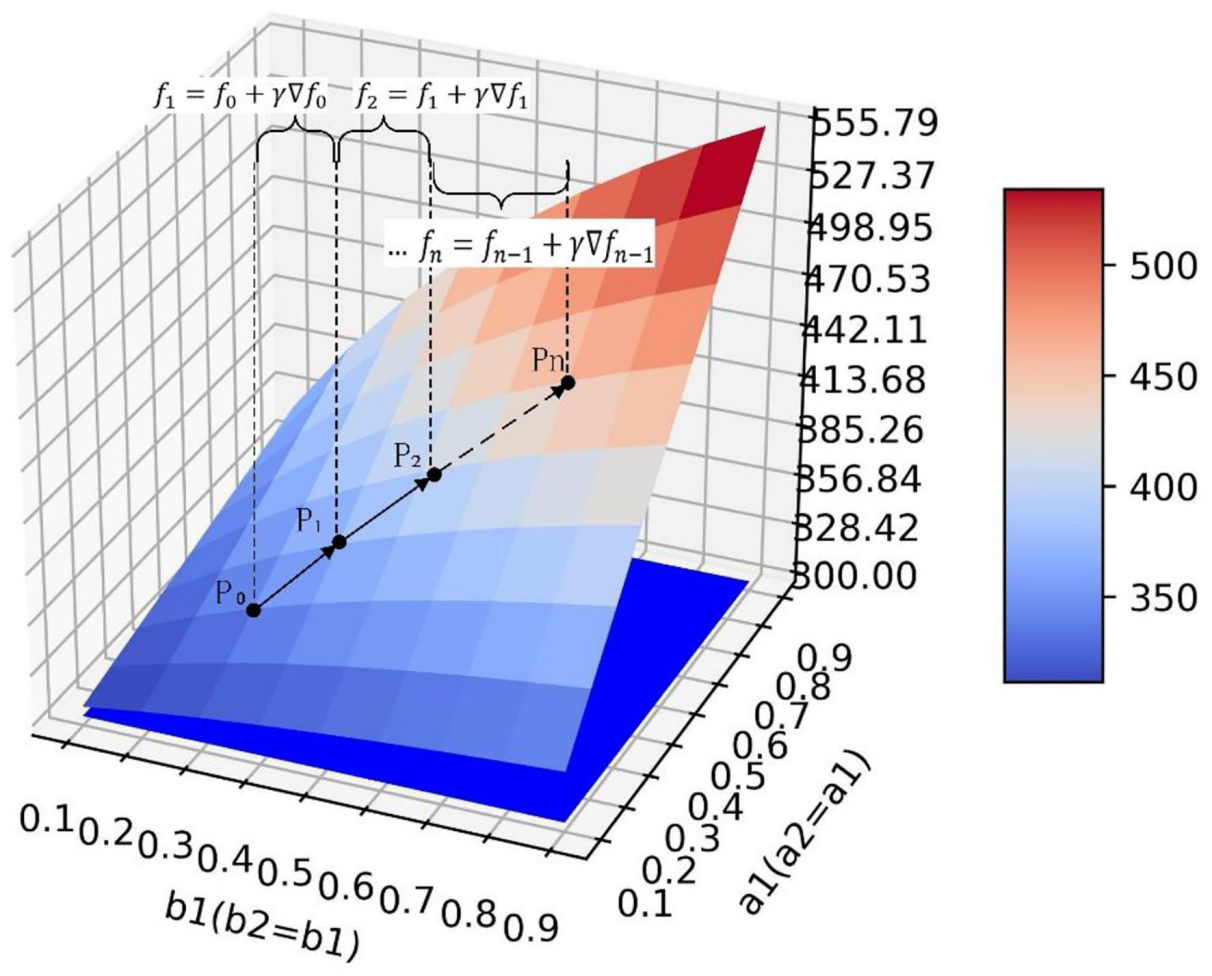
The optimal policy path on the surface.

In Equation (5), γ(γ > 0) is step length which defines how much the current parameter vector $\left(a_{1}^{<n>},\;b_{1}^{<n>}\right)$ changes along the gradient direction. Figure 13 shows the formation of the optimal policy path.

## Experiment

After natural disasters, accident disasters, social security incidents, and other emergencies, people's emotions tend to be negative. Effective positive guidance of people's emotions is an important content of social governance. Under the background of COVID-19 pandemic, people's emotional guidance in emergencies is facing new situations and challenges: First, the double superposition of epidemic risk and event risk has strengthened the breeding and dissemination of negative emotions among the people. In addition, due to the epidemic prevention and control, a large number of people's social activities and information dissemination have been transferred online, and guiding netizens' emotions through network channels has become a significant problem.

### Data Preparation

Since July 19, 2021, Zhengzhou City, Henan Province, was hit by rainstorms for many days, resulting in an urban flood, and the impact of the disaster continued until early August. During the same period, Zhengzhou was also in the normalization stage of epidemic prevention and control. At the end of July, new local COVID-19 cases appeared again in Zhengzhou, and then the number of cases continued to increase. In the context of the spread of the epidemic, the sudden rainstorm disaster had a secondary impact on people's normal life, and a large number of negative emotions gathered and spread on the Internet.

**Figure 14:**
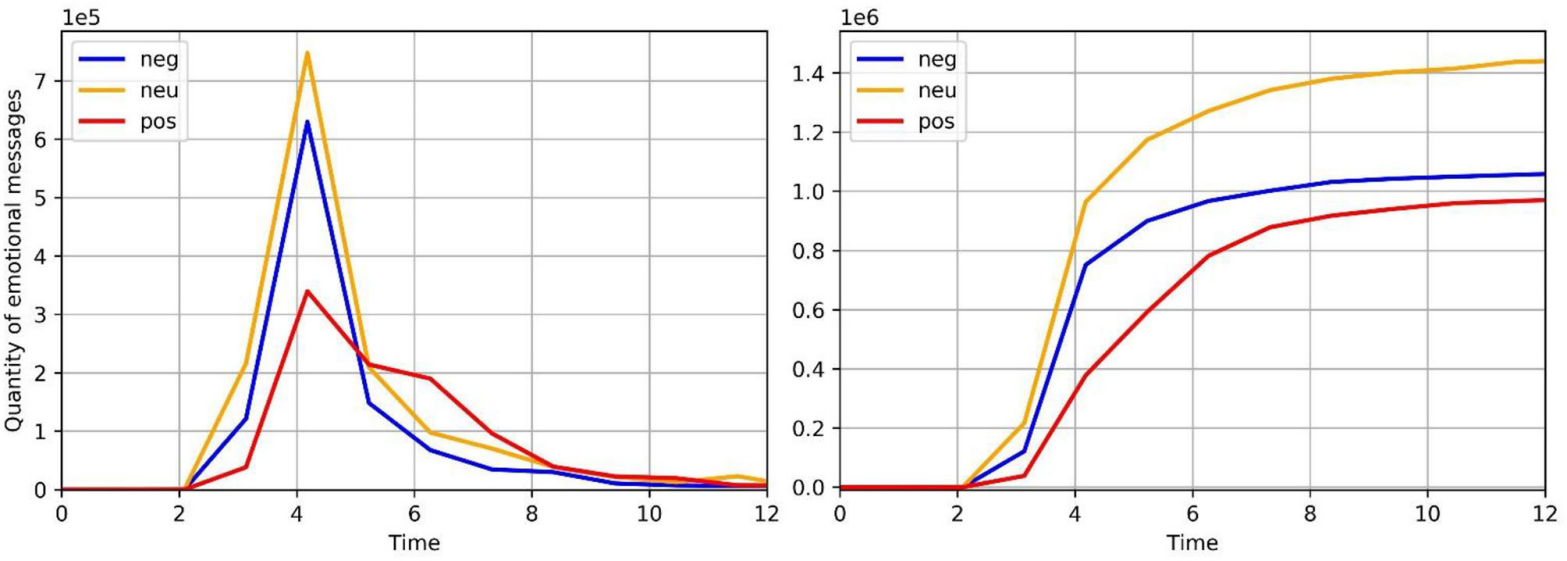
Amount of emotional information.

In the early stage of the flood situation, most netizens expressed anxiety about the extreme rainfall and concerns about the spread of the epidemic. Negative emotional information was dominant on the Internet. In response to this situation, relevant government departments took emergency measures in time to publish flood information and the situation of epidemic prevention and control, and actively guided online public opinion to reduce negative emotional information on the Internet. This study obtains emotional data through the network public opinion monitoring platform (https://www.gsdata.cn), which uses the natural language processing model (NLP) to divide the text information published by netizens into different emotions and publish the results on the platform. Therefore, we used this platform to collect the statistical data of positive information, neutral information, and negative information on the Internet from 19 July to 30 July with “Zhengzhou rainstorm” as the key word. It can be seen from Figure 14 that in the first 5 days, the amount of negative information and neutral information is far more than that of positive information. Starting on the fifth day, the amount of positive information began to outnumber the amount of negative and neutral information. This is a typical scene of emotion transfer from negative emotion and neutral emotion to positive emotion, indicating that the emergency guidance measures of relevant government departments played a positive role. Therefore, based on the collected data, we carried out an experiment.

### Model Constructing and Analysis

In the model, variables *x*_1_, *x*_2_, and *y* represent the amount of negative information, neutral information, and positive information, respectively. Various parameters of the model can be calculated by regression analysis introduced in Section Parameters Estimation (Table 6).

**Table 6:** Results of regression analysis.

**Parameters**	**Value**
*r*_1_	1.56996
*r*_2_	1.05991
*r*	1.16771
*K*_1_	1,570,565.10101
*K*_2_	2,245,255.63151
*K*	909,502.33298
*a*_1_	0.01
*a*_2_	0.25
*b*_1_	0.44308
*b*_2_	0.49794

The fitting result of the model is tested by *R*^2^, VIFs, and *F*-values. The results are as follows:


$$\{\begin{array}{c} R_{1}^{2}=0.96691,VIF_{1}=15.36421,F_{1}=1.99017\\ R_{2}^{2}=0.96507,VIF_{2}=14.56821,F_{2}=1.22290\\ R^{2}=0.99238,VIF_{3}=65.87175,F_{3}=1.01755 \end{array}$$

**Figure 15:**
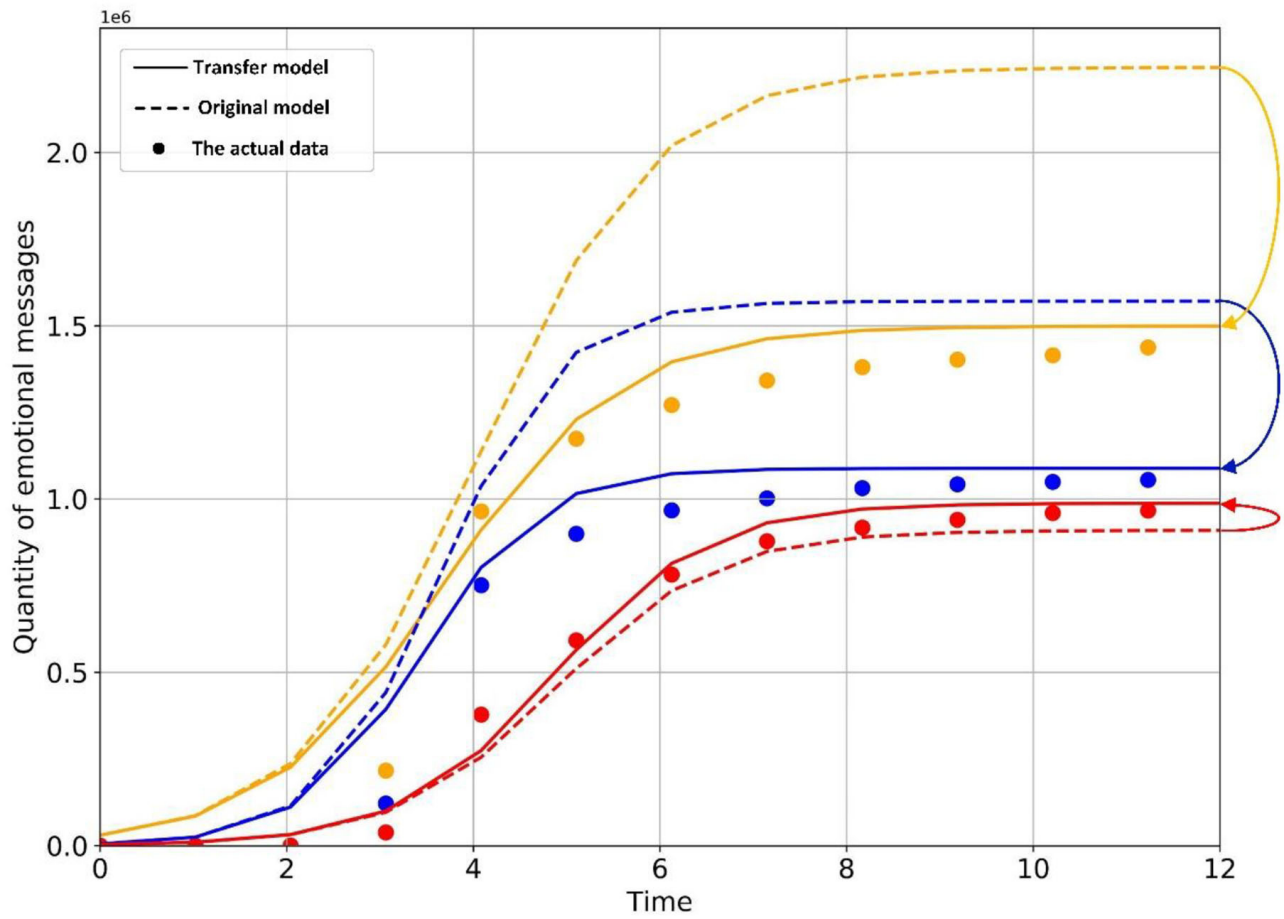
The actual data and fitting curves.

Plug parameters into the model and draw the graph of theoretical Transfer Model, Original Model, and actual data (Figure 15). It can be seen from the graph and R-Square that the theoretical model fits well and has research value. At the equilibrium point, the amount of negative emotion information decreased from 1,570,565.10101 to 1,088,345.87043, decreasing by 30.7%, the amount of neutral emotion information decreased from 2,245,255.63158 to 1,498,893.23150, decreasing by 33.2%, and the amount of positive emotion information increased from 909,502.33298 to 987,878.46469, increasing by 7.9%. According to the analysis of the data, the increase of positive emotion information is not large, which is far less than the decrease in negative emotion and neutral emotion. Relevant government departments need to adjust the guidance strength and efficiency to improve the guidance effect.

### Analysis of Guidance Strategy Optimization

The three-dimensional surface of positive emotional information is fitted by the least square method in four scenarios. The ŷ is the amount of positive emotional information and *n* is the number of iterations.

Scenario 1:


$$\{\begin{array}{c} \hat{y}=4.3847\times 10^{-5}b_{1}^{2}+865746.0065a_{1}b_{1}-297138.1949a_{1}^{2}\\ +126326.8251b_{1}+297138.1949a_{1}+859484.0702\\ \frac{\partial \hat{y}}{\partial a_{1}}=\;-\;594276.3897a_{1}+865746.0065b_{1}+\;297138.1948\\ \frac{\partial \hat{y}}{\partial b_{1}}=865746.0065a_{1}+8.7694\times 10^{-5}b_{1}+126326.8251\\ \gamma =1\times 10^{-7}\\ n=11 \end{array}$$

Scenario 2:


$$\{\begin{array}{c} \hat{y}=3.9480\times 10^{-5}b_{1}^{2}-0.0001a_{1}b_{1}-297138.1949a_{1}^{2}\\ +559199.8284b_{1}+297138.1948a_{1}+859484.0703\\ \frac{\partial \hat{y}}{\partial a_{1}}=\;-\;594276.3898a_{1}-0.0001b_{1}+\;297138.1948\\ \frac{\partial \hat{y}}{\partial b_{1}}=-\;0.0001a_{1}+7.8960\times 10^{-5}b_{1}+559199.8284\\ \gamma =1\times 10^{-7}\\ n=8 \end{array}$$

Scenario 3:


$$\{\begin{array}{c} \hat{y}=6.0666\times 10^{-6}b_{1}^{2}-0.0001a_{1}b_{1}-297138.1949a_{1}^{2}\\ -0.0001b_{1}+730011.1981a_{1}+922647.4829\\ \frac{\partial \hat{y}}{\partial a_{1}}=\;-\;594276.3897a_{1}-0.0001b_{1}+\;730011.1981\\ \frac{\partial \hat{y}}{\partial b_{1}}=1.2132\times 10^{-5}a_{1}-\;0.0001b_{1}-\;0.0001\\ \gamma =2\times 10^{-7}\\ n=10 \end{array}$$

Scenario 4:


$$\{\begin{array}{c} \hat{y}=-2.5266\times 10^{-6}b_{1}^{2}+865746.0065a_{1}b_{1}-297138.1949a_{1}^{2}\\ -432873.0034b_{1}-135734.8085a_{1}+1355520.4862\\ \frac{\partial \hat{y}}{\partial a_{1}}=\;-\;594276.3898a_{1}+865746.0065b_{1}-\;135734.8085\\ \frac{\partial \hat{y}}{\partial b_{1}}=865746.0065a_{1}-5.0532\times 10^{-6}b_{1}-\;432873.0034\\ \gamma =1\times 10^{-7}\\ n=11 \end{array}$$

**Figure 16:**
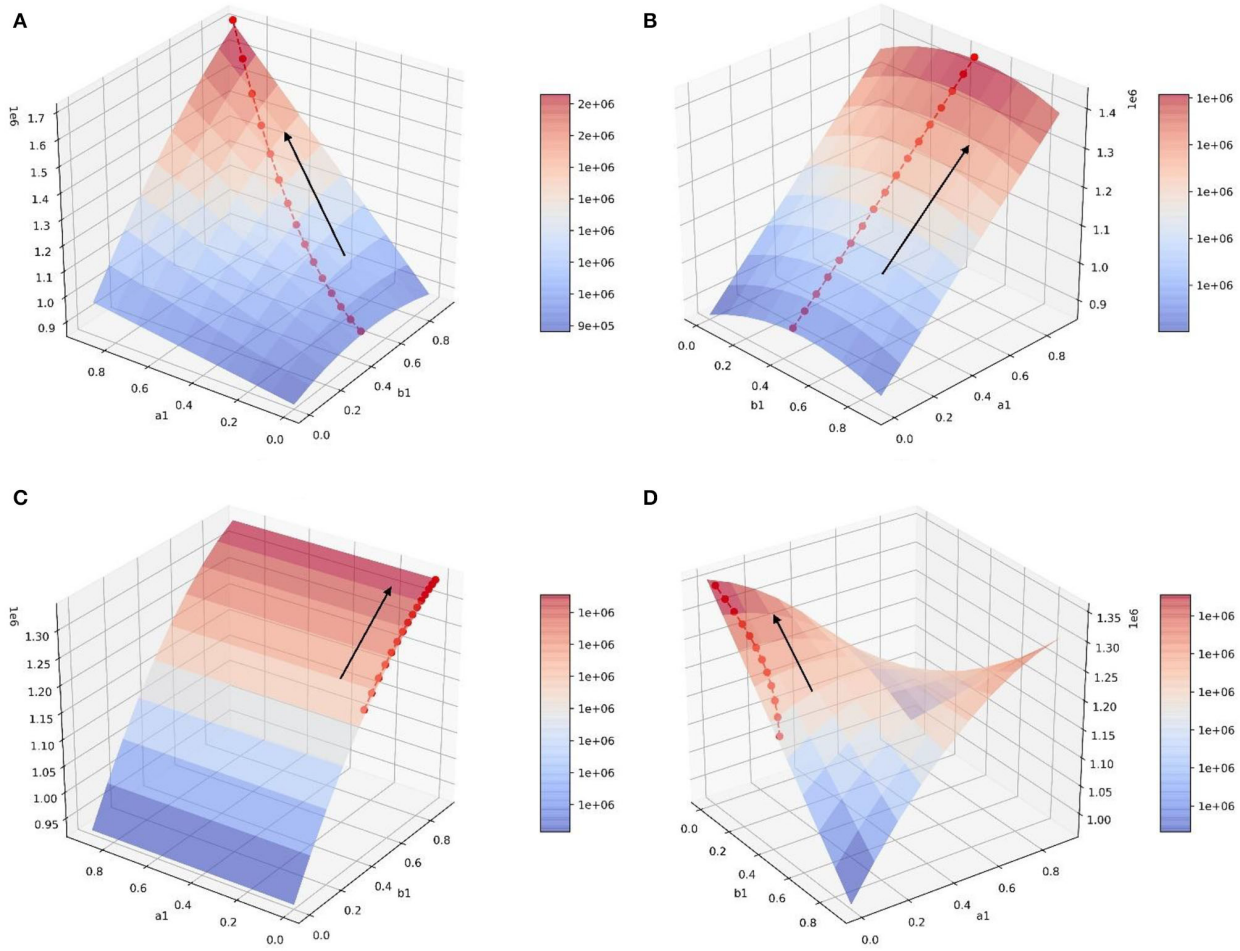
Optimal policy paths in four scenarios. **(A)** Optimal policy path in scenario 1. **(B)** Optimal policy path in scenario 2. **(C)** Optimal policy path in scenario 3. **(D)** Optimal policy path in scenario 4.

After *n* iterations of the gradient ascent method, the optimal policy path in each scenario can be obtained. In each scenario, the amount of positive emotional information at the balance point gradually increases along the strategy path (Figure 16), which obtains a better guidance effect and proves the effectiveness of the optimization strategy.

**Figure 17:**
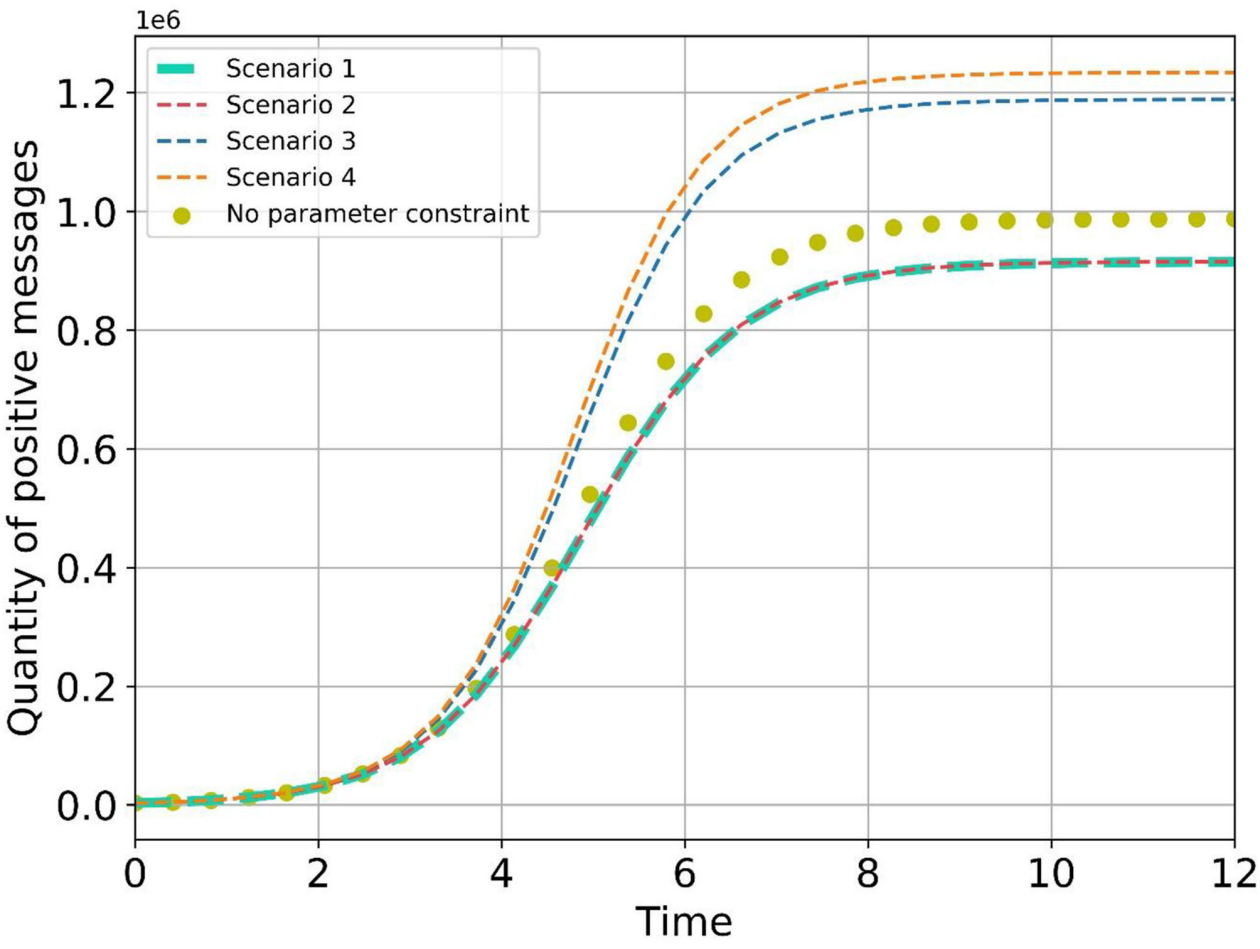
Evolution curves of positive information in different scenarios.

Due to the constraint of the numerical relationship between *a*_1_, *a*_2_, *b*_1_, and *b*_2_ in four hypothetical scenarios, we need to select a scenario that is more consistent with the data of this case. On the premise that the values of *r*_1_, *r*_2_, *r, K*_1_, *K*_2_, *K, a*_1_, *b*_1_ are fixed, let *n* = 0, and based on the transfer model, calculating the evolution values of positive emotional information in each scenario with and without the constraint of *a*_1_, *a*_2_,*b*_1_, and *b*_2_, drawing the evolution curve (Figure 17) and calculating the Euclidean distance between the two sets of data, as shown in Table 7.

**Table 7:** Euclidean distance between the two sets of data.

**Scenario**	**Euclidean distance**	**The normalized**
Scenario 1	305,759.90752	0.12171
Scenario 2	304,742.78522	0.12131
Scenario 3	849,022.96511	0.33796
Scenario 4	1,052,655.39206	0.41902

In scenario 1 and scenario 2, the Euclidean distance is smaller, indicating that this case is more applicable to scenario 1 and scenario 2. By further analyzing the value changes of *a*_1_, *b*_1_, and ŷ in this case under two scenarios (Tables 8, 9) could be obtained:

**Table 8:** Scenario 1.

***n***	**a_1_**	**b_1_**	** $\hat{\text{y}}$ **	**Δa_1_/a_1_**	**Δb_1_/b_1_**	** $\Delta \hat{y}/\hat{y}$ **
0	0.01	0.443075	937,904.9282	0.000%	0.000%	0.00%
1	0.060992	0.451738	964,634.7057	509.917%	1.955%	2.850%
2	0.112733	0.464366	992,954.0483	84.834%	2.795%	2.936%
3	0.165568	0.480818	1,023,495.568	46.867%	3.543%	3.076%
4	0.219828	0.500989	1,056,884.252	32.772%	4.195%	3.262%
5	0.275833	0.524811	1,093,756.524	25.477%	4.755%	3.489%
6	0.333901	0.552244	1,134,777.444	21.052%	5.227%	3.750%
7	0.394344	0.583279	1,180,656.759	18.102%	5.620%	4.043%
8	0.457474	0.617936	1,232,164.408	16.009%	5.942%	4.363%
9	0.523604	0.656258	1,290,145.997	14.456%	6.202%	4.706%
10	0.593052	0.698315	1,355,538.692	13.263%	6.409%	5.069%
11	0.666141	0.744201	1,429,387.951	12.324%	6.571%	5.448%
12	0.743203	0.794031	1,512,865.486	11.568%	6.696%	5.840%
13	0.824578	0.847945	1,607,288.833	10.949%	6.790%	6.241%
14	0.910622	0.906104	1,714,142.918	10.435%	6.859%	6.648%

**Table 9:** Scenario 2.

***n***	**a_1_**	**b_1_**	** $\hat{\text{y}}$ **	****Δ**a_1_/a_1_**	****Δ**b_1_/b_1_**	** $\Delta \hat{y}/\hat{y}$ **
0	0.01	0.443075	938,397.7541	0	0	0
1	0.06592	0.446458	969,779.2398	84.830%	0.758%	3.236%
2	0.12184	0.44964	1,001,147.92	45.896%	0.708%	3.133%
3	0.17776	0.452633	1,032,505.271	31.458%	0.661%	3.037%
4	0.23368	0.455448	1,063,852.6	23.930%	0.618%	2.947%
5	0.2896	0.458095	1,095,191.062	19.309%	0.578%	2.861%
6	0.34552	0.460585	1,126,521.68	16.184%	0.541%	2.781%
7	0.40144	0.462928	1,157,845.359	13.930%	0.506%	2.705%
8	0.45736	0.465131	1,189,162.899	12.227%	0.474%	2.634%
9	0.51328	0.467203	1,220,475.008	10.895%	0.444%	2.566%
10	0.5692	0.469152	1,251,782.311	9.824%	0.415%	2.501%
11	0.62512	0.470985	1,283,085.364	8.945%	0.389%	2.440%
12	0.68104	0.47271	1,314,384.657	8.211%	0.365%	2.381%
13	0.73696	0.474331	1,345,680.623	7.588%	0.342%	2.326%
14	0.79288	0.475857	1,376,973.645	7.053%	0.321%	2.273%
15	0.8488	0.477292	1,408,264.064	6.588%	0.301%	2.222%
16	0.90472	0.478641	1,439,552.179	6.181%	0.282%	2.173%

By analyzing Figures 16A,B and Tables 8, 9, it can be seen that in the process of gradient rise, the change rate of *a*_1_ in both scenarios is greater than that of *b*_1_, indicating that in this case, improving the guidance efficiency is the key to improving the guidance effect. The relevant government departments have invested enough force in the guidance process but ignored the efficiency in the implementation process. This results in the Barrel Effect, which makes the increase of positive emotional information much smaller than the decrease of negative emotion and neutral emotion. What is more, in scenario 1, since *b*_1_ and *b*_2_ can increase synchronously, a better guidance effect is finally achieved compared with scenario 2, which indicates that attention should be paid to both neutral and negative emotional people and the neglect of one kind of people will also cause a poor guidance effect.

## Conclusions and Discussion

### Conclusions

We discussed the emotion transfer mechanism of netizens, and established the emotion transfer model through a differential equation:


$$\{\begin{array}{c} \frac{dx_{1}}{dt}=r_{1}x_{1}\left(1-\frac{x_{1}}{K_{1}}-b_{1}\frac{x_{1}}{K_{1}}\right),x_{1}\left(0\right)=x_{10}\\ \frac{dx_{2}}{dt}=r_{2}x_{2}\left(1-\frac{x_{2}}{K_{2}}-b_{2}\frac{x_{2}}{K_{2}}\right),x_{2}\left(0\right)=x_{20}\\ .\\ .\\ .\\ \frac{dx_{n}}{dt}=r_{n}x_{n}\left(1-\frac{x_{n}}{K_{n}}-b_{n}\frac{x_{n}}{K_{n}}\right),x_{n}\left(0\right)=x_{n0}\\ \frac{dy}{dt}=ry\left(1-\frac{y}{K}+\sum_{i=1}^{n}a_{i}b_{i}\frac{x_{i}}{K_{i}}\right),y\left(0\right)=y_{0} \end{array}$$

This article defines two key parameters: transfer coefficient and conversion proportion. In the practical application of the model, the value of the transfer coefficient represents the guiding efficiency of relevant government departments in netizens' emotion guidance, and the value of conversion proportion represents the guiding strength. This study qualitatively discussed the influence of transfer coefficient and conversion proportion on the process of emotion transfer. The simulation results mainly support the following three conclusions:

First, when the guidance strength is certain, the adjustment of guiding efficiency can only change the final guiding effect to a small extent. At the same time, when the guiding efficiency of emotions with a larger intrinsic growth rate is higher, the guiding effect also tends to be better, which shows that the relevant government departments should pay more attention to the emotion with a higher intrinsic growth rate when guiding the netizens' emotions. Second, when the guidance efficiency is certain, the change of guidance strength has a great impact on the guidance effect, which shows that it is the key to investing enough force in the emotional guidance of netizens. Finally, the research shows that the adjustment of guidance efficiency and guidance strength is not blind, which needs to be discussed according to the situation. This article discusses four scenarios. In the ideal situation of scenario 1 (Figure 6), the guidance effect will be better with the improvement of guidance efficiency and guidance strength. However, in scenario 2 (Figure 8), blindly increasing the guidance strength of one of the emotions may bring a worse guidance effect. Similarly, in scenario 3 (Figure 9), blindly changing the guidance efficiency will not have a significant impact on the guidance effect. It can be seen that in the above two scenarios (scenarios 2 and 3), the aimless adjustment of guidance efficiency and guidance strength will make the investment unable to get a better reward. Saddle points appear in scenario 4 (Figure 11), which makes the situation more complex. It is difficult to give the adjustment of guidance efficiency and guidance strength through intuitive judgment. In order to solve the above problem, this study innovatively puts forward the optimization method of guidance strategy based on the gradient ascent algorithm, which theoretically shows that the guidance effect and guidance strategy are quantifiable.

In the subsequent empirical research, the theoretical model was fitted through the actual data to verify the rationality of the above theory and the feasibility of the method. The empirical study showed three aspects of the public opinion event. First, the discussion about this event tended to be negative. The model showed that the upper growth limit and intrinsic growth rate of netizens' negative emotion (*r*_1_ = 1.56996, *K*_1_ = 1570565.10101) were greater than those of netizens' positive emotion (*r* = 1.16771, *K* = 909502.33298). Negative emotion gathered in netizens, so it was urgent to guide netizens' emotions. Second, the guidance work of relevant government departments played a positive role in the emotional transfer of netizens, and from the fifth day after the occurrence of this public opinion event, the growing amount of positive emotional information in 1 day began to be more than that of the other two emotions. The equilibrium point of the model showed the effect of the work of relevant government departments. When the amount of information on the three emotions tended to be stable, the amount of positive emotional information increased by 7.9% and the amount of negative emotional information decreased by 30.7%. Finally, the analysis of the guidance strategy optimization showed that the guidance effect of this event was not in the best state, and left a lot to be desired. It also showed that the efficiency of relevant government departments in emotional guidance was low, which made the transfer coefficient of negative emotion (*a*_1_ = 0.01) and neutral emotion (*a*_2_ = 0.25) in the model obtain small values and affected the final guidance effect.

### Limitations and Prospects

In future research, we will improve the model and apply it to more complex scenarios. First, the emotion transfer model in this article only describes the scene of a one-way transfer from multiple emotions to one emotion, but in reality, there will be a situation where a single emotion will be transferred to a variety of emotions or the transfer between multiple emotions. Therefore, it is necessary to change the value range of parameters in the model (allowing the values of *a*_*i*_ and *b*_*i*_ to be <0) or introduce new parameters to make the model better describe the emotion transfer process in complex scenes. In addition, in the simulation of this article, it is a relatively simple way to divide emotion into positive emotion, neutral emotion, and negative emotion. In the existing fine-grained research, emotion is divided into 5 types and 7 types. Therefore, it is necessary to introduce more variables into the equation system and carry out simulation research on *n* kinds of emotion (*n* > 3).

## Data Availability Statement

The original contributions presented in the study are included in the article/Supplementary Material, further inquiries can be directed to the corresponding author.

## Author Contributions

YX was involved in the theoretical research, model construction, and the reviewing of the manuscript. ZW was involved in the data collection, data analysis, and the writing of the manuscript. ML was involved in the reviewing and revising of the manuscript. YL helped to build the mathematical model in his paper. All authors contributed to the article and approved the submitted version.

## Funding

This work was supported by the National Social Science Fund of China (Grant No. 20BXW074).

## Conflict of Interest

The authors declare that the research was conducted in the absence of any commercial or financial relationships that could be construed as a potential conflict of interest.

## Publisher's Note

All claims expressed in this article are solely those of the authors and do not necessarily represent those of their affiliated organizations, or those of the publisher, the editors and the reviewers. Any product that may be evaluated in this article, or claim that may be made by its manufacturer, is not guaranteed or endorsed by the publisher.
